# Perfluorocarbon Nanoemulsions with Fluorous Chlorin-Type Photosensitizers for Antitumor Photodynamic Therapy in Hypoxia

**DOI:** 10.3390/ijms24097995

**Published:** 2023-04-28

**Authors:** Minh Tuan Nguyen, Elizaveta V. Guseva, Aida N. Ataeva, Andrey L. Sigan, Anna V. Shibaeva, Maria V. Dmitrieva, Ivan D. Burtsev, Yulia L. Volodina, Alexandra S. Radchenko, Anton E. Egorov, Alexey A. Kostyukov, Pavel V. Melnikov, Nikolai D. Chkanikov, Vladimir A. Kuzmin, Alexander A. Shtil, Alina A. Markova

**Affiliations:** 1 Emanuel Institute of Biochemical Physics, Russian Academy of Sciences, 4 Kosygin Street, 119334 Moscow, Russia; tuantonyx@yahoo.com (M.T.N.); anna-shiba@mail.ru (A.V.S.); burtsevid@gmail.com (I.D.B.); radchenko.alexa@gmail.com (A.S.R.); ae.yegorov@gmail.com (A.E.E.); akostyukov@gmail.com (A.A.K.); vladimirkuzmin7@gmail.com (V.A.K.); 2 A.N. Nesmeyanov Institute of Organoelement Compounds, Russian Academy of Sciences, 28 Vavilov Street, 119991 Moscow, Russia; faftor.belyaeva@mail.ru (E.V.G.); asigan@yandex.ru (A.L.S.); nik-chkan@yandex.ru (N.D.C.); 3 Department of Faculty Surgery № 1, Pirogov Russian National Research Medical University, 1 Ostrovitianov Street, 117997 Moscow, Russia; aataeva97@mail.ru; 4 Blokhin National Medical Research Center of Oncology, 24 Kashirskoe Shosse, 115522 Moscow, Russia; dmitrieva.m@ronc.ru (M.V.D.); uvo2003@mail.ru (Y.L.V.); shtilaa@yahoo.com (A.A.S.); 5 M.V. Lomonosov Institute of Fine Chemical Technologies, MIREA—Russian Technological University, 86 Vernadsky Avenue, 119571 Moscow, Russia; melnikovsoft@mail.ru; 6 Institute of Cyber Intelligence Systems, National Research Nuclear University MEPhI, 31 Kashirskoe Shosse, 115409 Moscow, Russia

**Keywords:** perfluorocarbons, chlorins, nanoemulsion, tumor cells, hypoxia, phototoxicity, necrosis, photodynamic therapy

## Abstract

The efficacy of photodynamic therapy (PDT) strictly depends on the availability of molecular oxygen to trigger the light-induced generation of reactive species. Fluorocarbons have an increased ability to dissolve oxygen and are attractive tools for gas delivery. We synthesized three fluorous derivatives of chlorin with peripheral polyfluoroalkyl substituents. These compounds were used as precursors for preparing nanoemulsions with perfluorodecalin as an oxygen depot. Therefore, our formulations contained hydrophobic photosensitizers capable of absorbing monochromatic light in the long wavelength region and the oxygen carrier. These modifications did not alter the photosensitizing characteristics of chlorin such as the generation of singlet oxygen, the major cytocidal species in PDT. Emulsions readily entered HCT116 colon carcinoma cells and accumulated largely in mitochondria. Illumination of cells loaded with emulsions rapidly caused peroxidation of lipids and the loss of the plasma membrane integrity (photonecrosis). Most importantly, in PDT settings, emulsions potently sensitized cells cultured under prolonged (8 weeks) hypoxia as well as cells after oxygen depletion with sodium sulfite (acute hypoxia). The photodamaging potency of emulsions in hypoxia was significantly more pronounced compared to emulsion-free counterparts. Considering a negligible dark cytotoxicity, our materials emerge as efficient and biocompatible instruments for PDT-assisted eradication of hypoxic cells.

## 1. Introduction

Photodynamic therapy (PDT) has attracted attention as a minimally invasive method of cancer treatment. PDT is based on the interaction of the photosensitizer (PS), light and oxygen [1]. Upon irradiation with light at a specific wavelength, PS undergoes transition from the ground state S_0_ to the excited state S_1_; this is followed by intersystem crossing to the excited triplet state T_1_. Reactions with biomolecules produce reactive oxygen species (ROS, type I reactions); alternatively, molecular oxygen is converted into the highly reactive singlet oxygen ^1^O_2_ (type II reactions). Highly cytotoxic products of these photochemical reactions trigger the oxidative destruction of tumor cells. Consequently, the efficacy of PDT, being critically dependent on oxygen supply, is very limited in hypoxic tumors [2]. Moreover, PDT can aggravate the existing hypoxia, leading to tumor resistance to photoexcitation and disease progression [3].

One promising strategy to overcome tumor hypoxia is the use of perfluorocarbons (PFCs) as oxygen carriers [4]. PFCs are able to dissolve 20- to 40-fold larger amounts of oxygen compared to water. In addition, the lifetime of singlet oxygen in PFCs is 7000 times longer than in water, which is a serious advantage for PDT [5]. Therefore, incorporation of a PS into PFCs emerges as a promising approach to eliminate tumor cells in hypoxia [6]. To this end, three PFC-based strategies have been developed to enhance PDT efficacy via improved oxygen delivery, including the following: (1) synthesis of PS-loaded PFC nanoparticles [7,8,9]; (2) fluorinated polymers to encapsulate PS [10,11,12]; and (3) PFC nanoparticles with fluorous PS [6,13]. The first approach is simple and requires no organic synthesis, as all materials are commercially available. However, most PSs have poor compatibility with fluorous solvents, leading to leakage and premature release from the carriers [6]. The second approach presumes the use of fluorinated polymers that act both as surfactants and a fluorous phase for PSs. The third approach [6,13] utilizes a PS modified with perfluorinated alkyl chains to minimize aggregation and place the PS in a closer proximity to oxygen-enriched PFCs. However, porphyrin-based PSs used in these studies absorb light in the UV-A region, therefore tissue photodamage is limited. 

Chlorins are among the preferred PS scaffolds due to a strong absorption in the long wavelength region of visible light (600–700 nm), allowing for a deeper tissue photodamage [1,14,15,16]. In this study, we synthesized three fluorous chlorin-based PSs with different polyfluoroalkyl chains and loaded them into PFCs to form photoactive nanoemulsions (PFC-NEs). The term ‘fluorous’ was originally used analogously to ‘aqueous’ to describe the PFC medium/phase [17]. Over the years, the definition has evolved to mean ‘having characteristics of highly fluorinated saturated organic materials, molecules or molecular fragment’ [18,19]. The most distinctive feature of fluorous compounds is their high affinity to the fluorous phase (fluorophilicity) depending on the quantity and the length of perfluorinated non-polar fragments based on sp^3^-hybridized carbon [18]. Although fluorinated chlorins (FCs) have been successfully synthesized and extensively studied [15,20,21,22,23], most of the fluorinated substituents in these compounds were derivatives of hexafluorobenzene, which are not fluorous according to the above definition because the perfluoroaryl fragment is significantly more polar than perfluoroalkyl chains and the carbon atoms are sp^2^-hybridized. 

To our knowledge, the synthesis of chlorin derivatives with the fluorine content sufficient for proper fluorophilicity has not been reported. Therefore, this is the first study exploring the structure, physico-chemical properties and phototoxicity of fluorous chlorin-type PSs. Most importantly, PFC-NEs carrying PSs encapsulated into an optimized environment for oxygen-dependent photochemical reactions enhanced PDT efficacy in hypoxic cells. 

## 2. Results

### 2.1. Synthesis

**Scheme 1:**
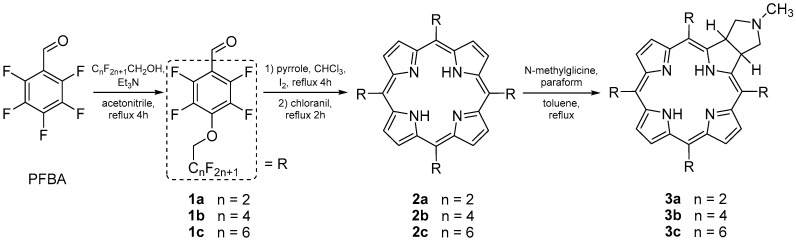
Synthesis of chlorins **3a**–**c**.

To obtain PSs for hypoxia, we decorated the periphery of the chlorin macrocycle with perfluoralkyl fragments. In preliminary experiments, we found that four fragments were minimally sufficient for the solubility in hydrophobic solvents (not shown). Linear perfluoralkyl fragments of different lengths (C2, C4 and C6) were used to compare the solubility of target compounds in perfluorodecalin (PFD), the main component of fluorocarbon emulsions. Compounds **3a**–**c** were prepared in three steps from commercially available reagents (Scheme 1). PFC fragments from fluorine-containing alcohols C_n_F_2n+1_CH_2_OH were introduced into the chlorin macrocycle at the first step by the nucleophilic substitution of the *para*-fluorine atom of pentafluorobenzaldehyde (PFBA). 

Preparation of benzaldehydes **1a**–**c** was carried out by reaction of PFBA with corresponding polyfluoroaliphatic alcohols. In our previous report, this reaction was performed in dimethylformamide (DMF) in the presence of potassium fluoride at room temperature for 5–10 h [13]. We optimized the reaction conditions and the method of isolation of the final product. Namely, the reaction was carried out by reflux in acetonitrile in the presence of triethylamine for 4 h. Then, the solvent was evaporated, and the residue was washed with sodium bicarbonate and extracted with hexane. The latter solvent provided the highest purity of target compounds compared to dichloromethane, chloroform and diethyl ether. This method of isolation provides sufficient purity of the final product for further transformations. Optimization of reaction conditions increased the product yield from 30% to 89%.

Previously, we have reported the synthesis of porphyrins **2a** and **2c** [13,24]. Scheme 1 shows an improved synthetic approach. Compounds **2a**–**c** were prepared by cyclization of benzaldehydes **1a**–**c** with pyrrole followed by oxidation with chloranil. We used the iodine catalysis for aldehyde–pyrrole condensation [25,26], but our procedure required heating of the reaction mixture without microwave activation. In comparison with the previous method in which the aldehyde–pyrrole condensation was carried out with boron trifluoride etherate [13], the iodine catalysis was advantageous due to a five-fold time shortening of the reaction. Additionally, the inert atmosphere was not needed, and the reaction components can be used at high concentrations (0.1 mol/L). Chloranil is a cheaper but not less efficient alternative to DDQ. Final products were obtained with 23–28% yields.

Finally, preparation of chlorins **3a**–**c** from porphyrins **2a**–**c** was carried out via [3+2]-cycloaddition of azomethine ylide (obtained from N-methylglycine and paraformaldehyde) to the porphyrin’s ‘quasi-isolated’ double bond [27]. Yields of **3a**–**c** were 30–65%.

### 2.2. Solubility

Generally, the solubility of organic compounds in fluorous solvents can be achieved with >50 weight % fluorine (wt% F) content [28,29]. Comparison of the solubility in PFD showed that, unlike porphyrins **2a**–**c** (45–56 wt% F), the solubility of chlorins **3a**–**c** (44–55% wt% F) strongly depended on the length of the PFC fragment. The solubility of **3b** and **3c** was as high as >100 mM compared to ~2 mM for **3a**. In contrast, the solubility of **2a–c** was ~1 mM. We explain this differential solubility by the presence of the N-methylpyrrolidine cycle outside of the macrocycle plane and by a smaller number of π-electrons that can reduce fluorophilicity [30]. Similar results have been reported for fluorous phthalocyanines (F-PCs) and subphthalocyanines (F-SubPCs). Non-planar F-SubPCs with a 14 π-electron system showed improved solubility in fluorous solvents compared to almost insoluble planar F-PCs with an 18 π-electron system despite nearly identical values of 52–59 wt% F [31]. These findings indicate that alteration of planarity improves drug solubility in fluorous solvents.

### 2.3. Formation and Characterization of PS Containing Emulsions

**Figure 1:**
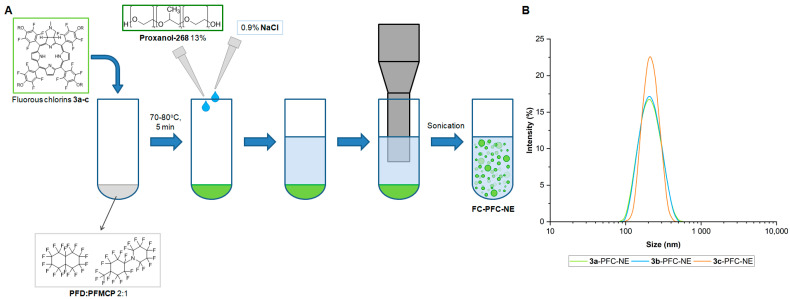
(**A**) Scheme of FC-PFC-NE preparation; (**B**) Size distribution of FC-PFC-NE determined by DLS.

FC-PFC nanoemulsions (FC-PFC-NEs) were prepared by ultrasonic emulsification. The nonionic block copolymer Proxanol-268 was used as a surfactant to stabilize NEs. According to dynamic light scattering (DLS), the average hydrodynamic diameter of resulting nanodroplets was ~200 nm (Figure 1). However, the atomic force microscopy showed that the size of particles was approximately twice smaller (Figure S2, Supplementary Materials). This difference is expected because DLS determines not only the particle size, but also ionic and solvent layers around the particle [32]. Changes in the droplet size and polydispersity index (PDI) of FC-PFC-NEs over 7 days at 4 °C were insignificant, suggesting that NEs are stable during this storage (Table 1). The droplet size remained ~200 nm after 30 days at −20 °C, allowing a long storage of frozen FC-PFC-NEs. The size of nanoparticles did not depend on the length of the polyfluoroalkyl substituent.

**Figure 2:**
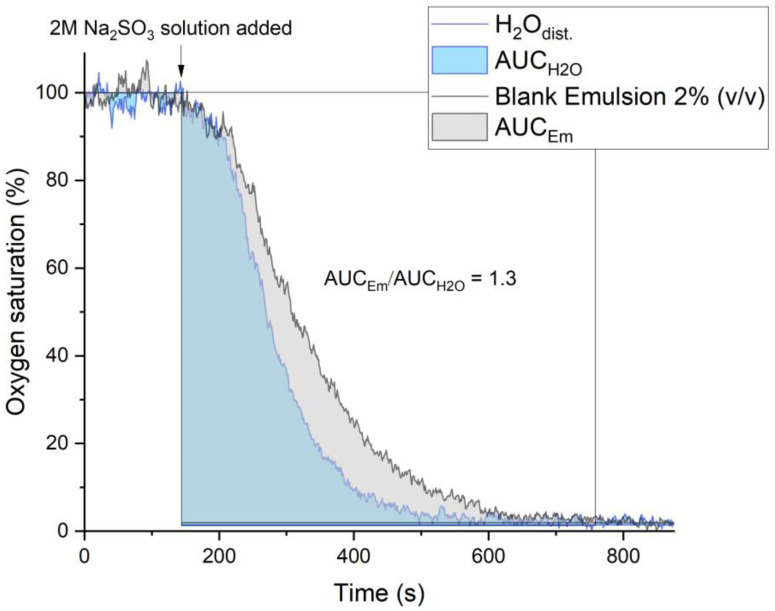
Time course of oxygen saturation in the aqueous medium with or without PFC-NE.

The oxygen capacity of PFC-NEs was determined using an optical oxygen analyzer Expert-009 (Econix-Expert, Moscow, Russia). The design and operation principles have been described earlier [33]. Water or 2% (*v*/*v*) PFC-NE saturated with atmospheric oxygen was placed in a Petri dish. An excess of sodium sulfite (to deplete molecular oxygen) was added after stabilization of sensor readings, and the time course of oxygen saturation in the medium was determined (Figure 2).

The PFD phase noticeably reduced the rate of oxygen depletion: the calculated area under the curve (AUC) increased by a factor of 1.3. Thus, PFC-NEs contain 1.3 times more O_2_ than water, which is consistent with literature data and our theoretical calculations (see Supplementary Materials, Section S2) [34,35].

### 2.4. Spectroscopy and Encapsulation Studies

**Figure 3:**
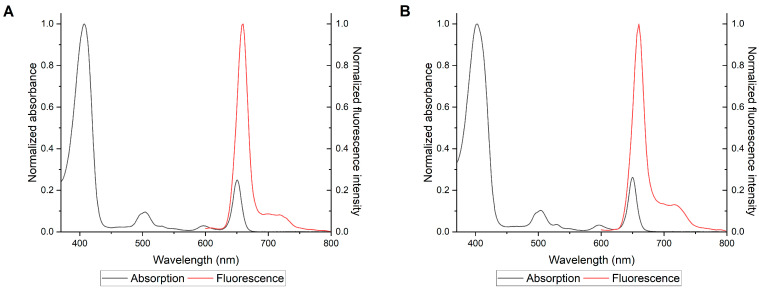
Normalized absorption and fluorescence spectra for **3b** in (**A**) DMF and (**B**) PFC-NE.

Absorption spectra of **3a**–**c** in DMF were typical for chlorin-type PSs, i.e., a major absorption band (Soret) and less pronounced Q-bands, of which an intense narrow long wavelength band is characteristic for chlorins (Figure 3, Figures S3 and S4). No significant changes in absorption and emission spectra of **3a**–**c** in PFC-NEs were observed (Table 2). The length of the polyfluoroalkyl substituent had no significant effect on the maxima of absorption bands (Table 2). Additionally, fluorescence and singlet oxygen quantum yield were not affected by the chain length, probably due to the remote distance from the macrocycle. All compounds showed a good balance between singlet oxygen generation and fluorescence in DMF with high values of Φ_Δ_ > 0.5 and Φ_F_ > 0.2, respectively (Table 2 and Table 3). Quantum yields of singlet oxygen suggest the potential of our modified chlorins as PSs in hypoxia.

The encapsulation efficiency of **3a**–**c** in PFC-NEs was determined by diluting the emulsions, centrifugation and subsequent spectrophotometric analysis. The second-derivative spectrophotometry was used because derivatization of absorption spectra eliminates the background signal from light scattering by NEs, thereby increasing the sensitivity and quality of measurements [36,37]. Data on the second-derivative spectra of **3a**-**cPFC-NE** and their supernatants (Figures S5–S7, Table S1 in Supplementary Materials) showed that an increased length of the polyfluoroalkyl substituent paralleled the decrease in encapsulation efficiency (Table 2). Thus, while the longer polyfluoroalkyl chains may facilitate dissolution in the fluorous phase, they may reduce the encapsulation, possibly due to incorporation into the surfactant micelles (proxanol-268).

### 2.5. Triplet States of ***3a–c***

**Figure 4:**
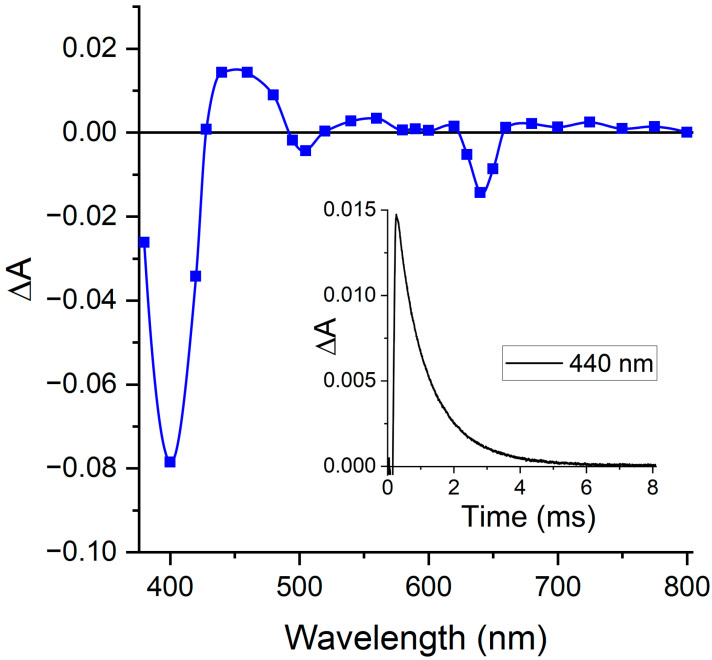
Transient absorption spectrum of **3b** (2.7 × 10^−7^ M) in DMF (300 μs after flash). Inset: kinetics of the triplet state decay.

Flash photolysis experiments were performed to investigate the triplet states of **3a**–**c**. Transient absorption spectra were recorded in DMF (Figure 4, Figures S8 and S9 in Supplementary Materials). 

Upon photoexcitation, the triplet absorption bands appeared in the regions 430–480 nm, 520–620 nm and 640–800 nm while singlet state bleaching was observed at Soret (380–428 nm), Q_y_ (490–510 nm) and Q_x_ (630–660 nm) bands (**3b** in Figure 4; Figures S8 and S9 for **3a** and **3c**, respectively), which is typical for chlorin derivatives [38]. The triplet state decays were monoexponential with a rate constant *k*_T_~1 × 10^3^ s^−1^ (Table 3). 

In the presence of molecular oxygen, the triplet state of PSs is rapidly quenched (Equation (1)):

(1) $$T_{1}{+}^{3}O_{2}\overset{k_{q}}{\to }S_{0}{+}^{1}O_{2}$$

Triplet state quenching with molecular oxygen in DMF was monitored using a laser flash photolysis setup. Oxygen solubility in DMF (2.0 × 10^−3^ M) was used as in [39]. The diffusion-controlled rate constant for DMF (η = 0.92 cP) was estimated to be *k*_diff_ = 8.3 × 10^9^ M^−1^ s^−1^ [40]. Considering the spin-statistical factor of 1/9, the resulting value is 9.2 × 10^8^ M^−1^ s^−1^. The calculated quenching constant is *k*_q_ = 1.1 × 10^9^ M^−1^ s^−1^, which is in a good agreement with the rate of the diffusion-controlled reaction. 

**Figure 5:**
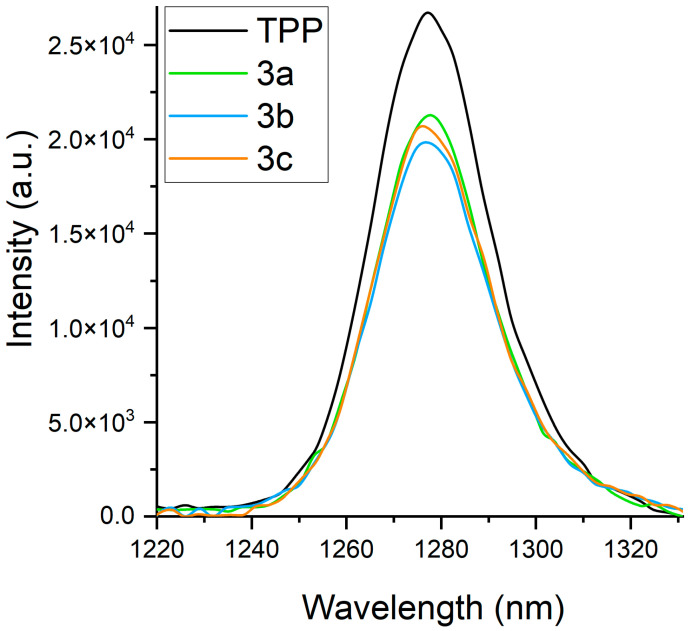
Spectra of singlet oxygen luminescence for **3a**–**c** and TPP. Excitation at 507 nm. Solvent, DMF.

Singlet oxygen is formed as a result of energy transfer from the excited triplet state to molecular oxygen (Equation (1)). Luminescence of ^1^O_2_ can be detected in the near-infrared region (λ_max_ = 1275 nm) (Figure 5).

Determination of singlet oxygen quantum yields for **3a–c** revealed both an efficient intersystem crossing from S_1_ to T_1_ and an efficient energy transfer to molecular oxygen (Φ_Δ_~0.5) (Figure 5). 5,10,15,20-tetraphenylporphyrin (TPP) was used as a reference (Φ_Δ_ = 0.64 in DMF [41]). Comparison with similar N-methyl pyrrolidine-fused *meso*-5,10,15,20-tetrakis(pentafluorophenyl)chlorin (Φ_Δ_ = 0.64 in DMF [42,43]) showed that the substitution of *para*-fluorine atoms in *meso*-pentafluorophenyl groups by polyfluoroalkoxy moieties did not impact the singlet oxygen generation (Table 3). Importantly, our modification for PFC-NE formulation did not alter PS-related characteristics of synthesized **3a–c**.

### 2.6. Intracellular Accumulation of FC-PFC-NEs

**Figure 6:**
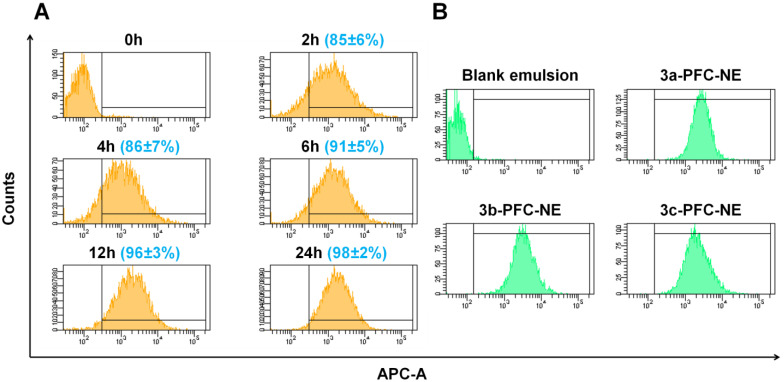
**FC-PFC-NE** accumulation in HCT116 cells determined by flow cytometry. (**A**), Time course of cell-associated fluorescence after loading with **3b-PFC-NE.** Percentages of positive (drug accumulated) cells are given in parentheses as mean ± SD. (**B**), **3a-c-PFC-NE** uptake at 24 h. Shown are representative experiments (*n* = 3).

Intracellular accumulation was assessed by flow cytometry taking advantage of fluorescence of chlorin-containing emulsions. Already after 2 h of loading, ~85% of HCT116 cells accumulated **3b-PFC-NE**. Maximum accumulation was reached within 12 h and remained unchanged at the 24 h mark (Figure 6A). PFC-NEs containing **3a**, **3b** or **3c** showed a similar pattern of accumulation (Figure 6B). In further experiments, we exposed the cells to **FC-PFC-NEs** for 24 h followed by light illumination. 

### 2.7. Colocalization with Intracellular Trackers 

**Figure 7:**
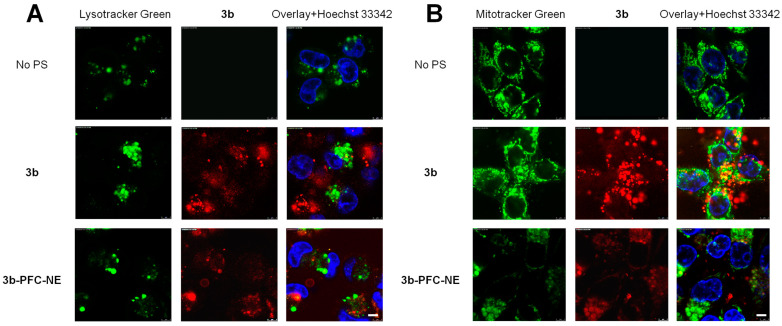
Distribution of **3b** and **3b-PFC-NE** (10 μM chlorin each) in HCT116 cells. (**A**), Colocalization with LysoTracker Green, (**B**), Colocalization with MitoTracker Green. Cells were loaded with the indicated compounds for 24 h, washed, stained with intracellular dyes and analyzed by confocal laser scanning microscopy. Fluorescence signals: green—Lyso- or MitoTracker Green, red—**3b**, blue—Hoechst 33342. Bar, 5 μm.

To analyze intracellular distribution, we chose **3b** as the most synthetically available FC. Additionally, **3b** showed a good solubility in PFCs, a reasonable degree of encapsulation in FC-PFC-NEs and an acceptable average lifetime of the triplet state. Confocal laser scanning microscopy experiments were performed to study the colocalization of emulsion-free **3b** (dissolved in DMF) or **3b-PFC-NE** with Hoechst 33342 (a nuclear dye) and LysoTracker Green or MitoTracker Green (lysosomal and mitochondrial dyes, respectively). Cells were incubated with **3b** or **3b-PFC-NE** (each at 10 µM chlorin) for up to 24 h and washed with saline before staining. Figure 7 shows the pattern of intracellular distribution after 24 h, a time interval at which intracellular accumulation reached plateau (Figure 6). The emulsion-free **3b** localized in lysosomes and mitochondria. The **3b-PFC-NE** accumulated largely in mitochondria and was slightly discernible in lysosomes. Neither compound was detectable in the nuclei (Figure 7). Earlier time intervals are presented in Figure S10.

### 2.8. FC-PFC-NEs in Cellular PDT

To test the photodamaging potency of FC-PFC-NEs, HCT116 colon carcinoma, MCF7 breast carcinoma cell lines or non-malignant fibroblasts were loaded with 1.5–15 μM of **3a**–**c** (dissolved in DMF) or the respective FC-PFC emulsions. The clinical PS Photolon, a trisodium salt of chlorin e6, served as a positive control for photodamage [44]. Cell death in hypoxia was studied in two settings: a) cells were cultured for 8 weeks in the incubator with O_2_ displaced with N_2_ (1% O_2_, 5% CO_2_) (prolonged hypoxia) followed by photoexcitation in normoxia (20.9% O_2_, 5% CO_2_), and b) cells cultured in normoxia were illuminated at 1% oxygen. The photoinducible cytotoxicity was studied after loading with **3a**–**c**, **3a-cPFC-NE** or Photolon for 24 h (to achieve maximal intracellular accumulation) followed by removal of the medium and illumination of cell monolayers with a 660 nm, 45 J/cm^2^ laser under normoxic or hypoxic conditions. After photoexcitation, cells were incubated for an additional 24 h under the respective conditions as during illumination. Control cells were loaded with the respective amounts of vehicles (DMF or blank FC-free emulsion).

Table 4 summarizes the results of cellular PDT depending on oxygen content. Most importantly, **3a-cPFC-NE** induced strong photodamage of HCT116 and MCF7 cells cultured either in normoxia or in hypoxia prior to illumination. IC_50_ values were within the submicromolar-to-low micromolar range of concentrations. Each individual NE was superior over Photolon. In striking contrast, the emulsion-free **3a–c** preparations were significantly less potent (Figure S11). These results confirm the critical role of PFCs as a solvent for polyfluoroalkylated PSs: in the aqueous phase, the intracellular fluorescence of **3a–c** can be observed (Figure 7), although no lethal cell photodamage is achievable. Moreover, the increased oxygen capacity and deposition of oxygen in the fluorous phase of NEs significantly improved PDT efficacy both in normoxia (compared to Photolon) and in hypoxia. Interestingly, **3a-cPFC-NE** were less efficient PS in non-malignant fibroblasts (Table S2). Dark cytotoxicity after 48 h of incubation with each FC-PFC-NE was negligibly low (IC_50_ > 15 µM).

### 2.9. FC-PFC-NEs Trigger Rapid Photonecrosis: A Key Role of Nanoformulations in Hypoxia

**Figure 8:**
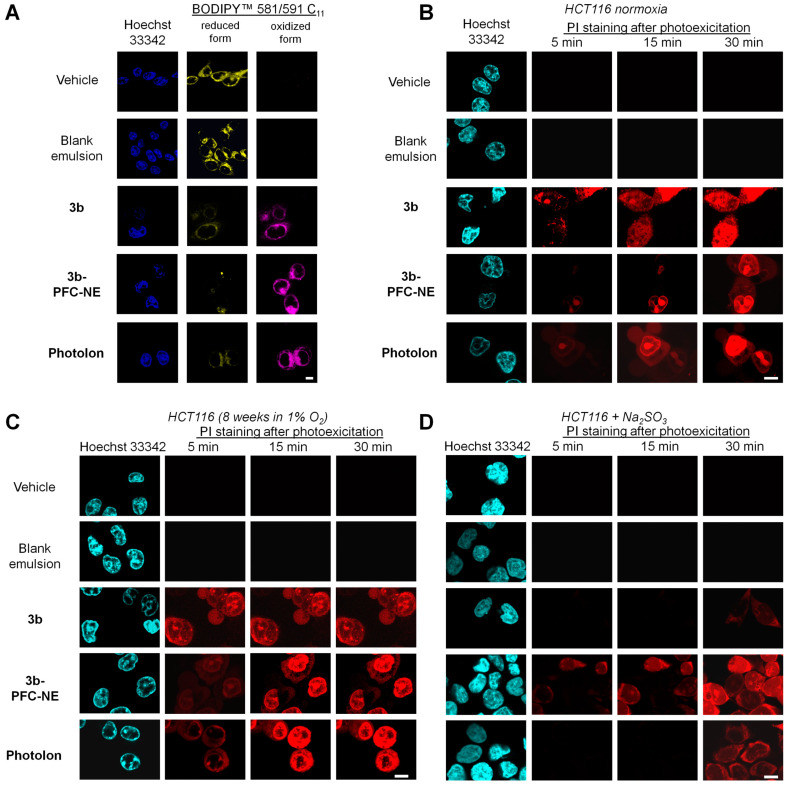
Efficacy to trigger photonecrosis depends on oxygen content and PS formulation. The HCT116 cells were loaded with indicated compounds (each 10 µM chlorin) for 24 h. BODIPY™ 581/591 C11 (yellow—reduced form, magenta—oxidized form), Hoechst 33342 (blue, cyan) and PI (red) were added, and cells were illuminated with a light source (405 nm). ***Top*** *panel*: normoxia. (**A**), lipid peroxidation; (**B**), PI fluorescence. ***Bottom*** *panel*: PI fluorescence in hypoxic cells. (**C**), prolonged; (**D**), acute. Bar, 5 μm.

To characterize the mechanism of photoinduced cell death, HCT116 cells were incubated with the emulsion-free **3b** or with **3b-PFC-NE** (each at 10 μM chlorin) for 24 h prior to illumination by the confocal microscope laser in the Soret band (405 nm). We analyzed lipid peroxidation of illuminated cells by fluorescence of reduced vs. oxidized forms of the BODIPY™ 581/591 C11 sensor. Increased fluorescence of the oxidized form indicates lipid peroxidation that precedes the plasma membrane damage. A more pronounced lipid peroxidation was observed with **3b-PFC-NE** compared to **3b** (Figure 8A). 

Propidium iodide (PI), a marker of the plasma membrane integrity [45,46], was added to the culture medium prior to illumination. As shown in Figure 8B, nuclear fluorescence of this DNA intercalator was readily detectable within the initial minutes post illumination. These data indicated the necrotic mode of cell photodamage [21,24]. To investigate whether **3b** and **3b-PFC-NE** retain the ability to trigger photonecrosis under hypoxic conditions, HCT116 cells were cultured in hypoxia for 8 weeks (prolonged hypoxia) followed by illumination in air for the analysis of the death mechanism as described above for normoxia. Alternatively, HCT116 cells were treated with 2 mM sodium sulfite to induce acute hypoxia and illuminated in the presence of this agent. Similarly to normoxia, we registered rapid PI entry upon cell illumination after prolonged hypoxia (Figure 8B): by 5–10 min the nuclei demonstrated PI-associated fluorescence. However, after the photoexcitation of cells in the presence of sodium sulfite, PI fluorescence was detectable within 5–15 min only in samples with **3b-PFC-NE**, whereas the emulsion-free **3b** evoked little or no effect. In hypoxic cells (sodium sulfite) illuminated in the presence of the reference PS Photolon, a weak cytoplasmic (RNA), but not nuclear, staining was detectable as late as 30 min post illumination. However, if cells were exposed to prolonged hypoxia and then illuminated under normoxic conditions (i.e., in air), the PS activity of Photolon was retained (compare the timing of PI fluorescence in Figure 8C,D). In other words, the potency of Photolon strongly depended on oxygen content, whereas the photoactivation of the oxygen-enriched **3b-PFC-NE** was similarly cytocidal in normoxia and hypoxia. Overall, FC-PFC-NE formulations emerge as particularly applicable PSs for lethal damage of hypoxic cells via rapid photonecrosis.

## 3. Discussion

Tumor hypoxia dramatically limits the efficacy of PDT. The strategy of using PFCs for combined delivery of PSs and oxygen has been extensively explored [7,11,47,48,49]. Fluorinated PSs have been studied for photostability, ROS generation and pharmacokinetic profiles [20,23]. However, the examples of fluorous (i.e., soluble in PFC) PSs are scarce and are confined largely to porphyrin-based compounds whose photoactivation requires a shorter wavelength light with poor tissue penetration [6,13,24,50]. By modifying our previous series of fluorous porphyrins [13,24] using a 1,3-cycloaddition reaction, we obtained a novel type of fluorous chlorin-based PS with red light (650 nm) absorbance.

Hydrophobic PSs tend to aggregate in the aqueous phase, which limits their PDT efficacy. Incorporation of the hydrophobic PSs into chemically inert organic solvents such as PFCs allows to decrease aggregation and improve the photoactivating potency [51]. However, PFCs are exceptionally hydrophobic and at the same time lipophobic [52]. Therefore, an appropriate formulation is required for biological applications of PFCs.

Aiming at preparation of efficient and biocompatible materials for PDT in hypoxia, we synthesized fluorous chlorin-based PSs **3a–c** with hydrophobic tails of different lengths. Our results showed that compounds with as little as 44 wt% F (**3a**) can be dissolved in PFCs, allowing the synthesis of simpler fluorous molecules with a smaller fluorine content for biological applications.

Encapsulation of these compounds into PFCs produced ~200 nm **3a-cPFC-NE** droplets. This size is optimal for antitumor drug delivery considering the enhanced permeability and retention effect [53]. Encapsulation allowed to obtain reasonably large amounts of chlorin inside the droplets. Furthermore, **3a-cPFC-NE** contained larger amounts of oxygen than **3a–c**. Finally, the resulting NE remained stable at 4 °C and −20 °C for at least 30 days.

Compounds **3a–c** and **3a-cPFC-NE** demonstrated a number of characteristics relevant to the efficacy of PDT. These compounds readily entered human tumor cells and accumulated in the cytoplasm. Compound **3b** accumulated largely in mitochondria. Apparently, PS localization in this organelle can be deleterious for the fate of illuminated cells. Light activation of cells loaded with new compounds triggered rapid (within the initial minutes) lipid peroxidation followed by photonecrosis. Compounds **3a–c** and **3a-cPFC-NE** evoked a negligible dark cytotoxicity. Together, these results proved the applicability of new compounds in conventional (that is, normoxic) PDT.

Most importantly, our new **3a-cPFC-NE** formulations were superior over the emulsion-free **3a–c** in cell photodamage under hypoxic conditions. The phototoxic potency of NE in normoxia was similar to that of the clinical chlorin-based PS Photolon. In cells cultured at low oxygen and illuminated in air, **3a–c** and Photolon were potent PSs. In striking contrast, if cells were treated with sodium sulfite (depletion of molecular oxygen) and illuminated under the same hypoxic conditions, only **3a-cPFC-NE** demonstrated the ability to rapidly trigger the necrotic cell death. Thus, new nanoformulations can serve as a depot of oxygen. Delivered to the hypoxic cells, these materials provide sufficient oxygen supply for the efficacious PDT to achieve irreparable cell damage. 

Photonecrosis as an outcome of PDT has been achieved with a variety of tetrapyrrolic PSs. Modifications at the periphery of the macrocycle including one or more boron clusters, polyfluorination or conjugation of heterocyclic moieties did not alter the phototoxic potency [21,54,55]. In our chlorin-based NE, valuable characteristics of the initial PS, such as absorption in the long wavelength spectral region as well as high quantum yield of singlet oxygen upon light activation, were retained. Therefore, the micelles that incorporate hydrophobic chlorin derivatives, dissolved in the oxygen-enriched solvent, combine a number of important properties, namely, the nanoscale size, the photochemically advantageous PS and the oxygen depot. These materials are particularly beneficial in tumor hypoxia, a clinically unfavorable situation that seriously limits the efficacy of PDT and ionizing radiation, the treatments that presume sufficient amounts of tissue oxygen [56]. Moreover, in hypoxic tumors, certain mechanisms that implement the chemotherapeutic stress may be non-functional as a result of cell selection for survival in low oxygen [57]. In this scenario, photonecrosis as direct irreparable damage of membranes by lipid peroxides in response to light activation becomes a method of therapeutic choice for circumvention of hypoxia-associated pleiotropic resistance of tumor cells.

## 4. Materials and Methods

### 4.1. Synthesis of FCs

Synthesis of **2a–c** is presented in Supplementary Materials. To prepare **3a–c**, compounds **2a–c** (0.10 g, 1 eq.) were dissolved in 25 mL toluene. N-methylglycine (2 eq.) and paraformaldehyde (4.7 eq.) were added to these solutions, and the mixtures were refluxed for 5 h under argon atmosphere. The reaction was monitored by TLC. Additional portions of N-methylglycine (2 eq.) and paraformaldehyde (4.7 eq.) were added depending on the reactivity of substrates, and the mixture was refluxed for another 5 h. The solvent was evaporated under reduced pressure. Residues were purified by column chromatography on silica gel with gradient elution using CHCl_3_-ethyl acetate to yield **3a**–**c** (30–65%).

### 4.2. Solubility in PFD

Dissolution of **2a–c** and **3a–c** in PFD was carried out with heating. Solutions were kept at room temperature for 3 days to equilibrate. Saturated solutions were diluted for the measurement of optical density (0.1–1 units). Concentrations of **2a–c** and **3a–c** in solutions were calculated using known extinction coefficients in PFD (Supplementary Materials, Section Synthesis). The Qy(1,0) band in the absorption spectra was taken as an analytical wavelength for each compound.

### 4.3. Preparation and Characterization of PFC-NEs

Emulsions were prepared by an ultrasonication method using a Sonicator W-375 (Heat Systems-Ultrasonics, Plainview, NY, USA) [24]. The fluorous phase (F-phase) was prepared by mixing PFD and perfluoromethylcyclohexylpiperidine *w*/*w* 2:1. Stock solutions of **3a–c** in the fluorous phase (F-solutions, 0.5 mM) were prepared by dissolving 7.76 mg, 9.76 mg or 11.76 mg of **3a**, **3b** or **3c**, respectively, in 10 mL F-phase. To accelerate the dissolution, F-solutions were heated at 70–80 °C for 5 min. Proxanol-268 was pre-dissolved in saline (13%). To prepare PFC-NEs, 0.667 mL F-solution (or F-phase in case of blank emulsion, control), 2.05 mL 13% Proxanol-268 solution and 7.28 mL saline were mixed in a 20 mL tube. Sonication was carried out in a pulsed mode at 50% duty cycle. The power was set to 5. The procedure was performed on ice for 3 min and repeated four times with 1–2 min intervals to prevent sample heating. The hydrodynamic diameter of resulting emulsions was analyzed by DLS using a Zetasizer Nano ZS (Malvern Instruments Ltd., Malvern, UK). Additionally, the particle size was studied by atomic force microscopy on a Nanoscope V Multimode (Bruker, Billerica, MA, USA).

Oxygen content in the aqueous medium in the absence or presence of 2% (*v*/*v*) blank emulsion was analyzed by optical oximetry on an Expert-009 analyzer (Econix-Expert, Moscow, Russia) [33,58]. The method determines the phase shift *ϕ* between the modulated excitation light (525 nm) and the received phosphorescence signal, which is converted into the excited state decay time τ according to the equation:

(2) τ=tan(ϕ)/2πf,

where *f* represents the modulation frequency. Pt(II) 5,10,15,20-tetrakis (2,3,4,5,6-pentafluorophenyl)-porphyrin (PtTFPP, Frontier Scientific, Newark, DE, USA) served as an indicator dye encapsulated in a composite material. The fabrication procedure has been described in detail [59,60,61].

An amount of 4 ml of air-equilibrated water or 2% (*v*/*v*) PFC-NE was placed in a 60 mm Petri dish with the optical oxygen sensor composite material. After stabilization of sensor readings, the molecular oxygen was depleted by adding 2 mL 0.67 M sodium sulfite. The time course of oxygen content in the medium was determined.

### 4.4. Absorption and Fluorescence Spectroscopy

UV-Vis absorption spectra of **3a**–**c** in DMF and PFC-NEs were recorded using a UV-3101RS spectrophotometer (Shimadzu, Japan) in a 1 × 1 cm quartz cuvette at room temperature. To address the background scattering in emulsion samples, a mathematical method of correction of optical density was developed (Supplementary Materials, Section S3, Figure S1). Fluorescence spectra were obtained on a Fluorat-02 Panorama spectrofluorometer (Lumex, Russia).

Fluorescence quantum yields of **3a**–**c** were recorded in DMF and PFC-NEs using TPP as a standard (Φ_F_ = 0.10 in toluene [62]) at the excitation wavelength of 525 nm. Absorbance at the excitation wavelength was kept below 0.05/cm to avoid the inner filter effect (A = 0.03 for samples and standard).

Quantum yields of fluorescence were determined as described [63] according to the formula:

(3) $$\Phi_{f}^{i}=\frac{F^{i}f_{s}n_{i}^{2}}{F^{s}f_{i}n_{s}^{2}}\Phi_{f}^{s},$$

where $\Phi_{f}^{s}$ is the fluorescence quantum yield for the standard, $F_{i}$ and $F_{s}$ are integrated fluorescence intensities for the sample and the standard, $n_{i}^{2}$ and $n_{s}^{2}$ are squares of refractive indices in the solvent for the sample and the standard, respectively. $f_{i}=1-10^{-A_{x}}$, where A_x_ is sample absorption at the registration wavelength.

Singlet oxygen quantum yields Φ_Δ_ for **3a–c** in DMF were determined from singlet oxygen luminescence. TPP (Φ_Δ_ = 0.64 in DMF [41]) was used as a standard.

The luminescence spectra of singlet oxygen in DMF (λ_max_~1270 nm) were obtained by illumination with a Xe-lamp (photoexcitation at λ = 504 nm of air-equilibrated solutions at room temperature).

Quantum yields of singlet oxygen were determined in DMF according to the formula [64]:

(4) $$\Phi_{\Delta }^{i}=\frac{F^{i}(1-10^{-As})}{F^{s}(1-10^{-Ai})}\Phi_{\Delta }^{s},$$

where $\Phi_{\Delta }^{s}$ is the quantum yield of singlet oxygen for the standard, $F_{i}$ and $F_{s}$ are areas under the luminescence spectra of singlet oxygen for the sample and the standard, respectively, $A_{i}$ and $A_{s}$ are absorbances of the sample and the standard, respectively.

### 4.5. Flash Photolysis

The transient triplet–triplet absorption spectra were measured using a conventional flash photolysis setup (optical path length 20 cm). Excitation was performed with Xe lamp through red filters with transmission >620 nm, 80 J/15 µs. Signals were recorded by a PMT-38 photomultiplier (MELZ, USSR) at 380–800 nm. Solutions were degassed with the vacuum pump before the experiments.

Laser pulse photolysis was used to study triplet state quenching by molecular oxygen in air-equilibrated DMF solutions of **3a–c**. Experiments were performed on an LKS 80 (Applied Photophysics, Leatherhead, UK) instrument. The third harmonics of a Brilliant B Nd-YAG laser (Quantel, Lannion, France) were used for excitation. The wavelength in the range of 410–600 nm was tuned by an OPO (MagicPrism, OPOTEK Inc., Carlsbad, CA, USA). Kinetics of transient species generated by a 5 ns laser excitation pulse were registered by absorbance changes within 400–750 nm using a 150 Xe arc lamp. The detection system was equipped with a 600 MHz oscilloscope (Agilent Infiniium 10 GS/s) and an R928-type PMT.

### 4.6. Encapsulation Efficiency

The encapsulation efficiency was defined as the percentage of the PS entrapped into nanodroplets. To determine the encapsulation efficiency of a PFC-NE, we diluted the emulsion with water to a PS concentration of 10 uM. To separate heavy PFC nanodroplets (*p* = 1.95 g/cm^2^), samples were centrifuged for 15 min at 4000× *g*. Absorption spectra of initial samples and their supernatants were measured on a Benchmark Plus Microplate Reader (Bio-Rad Laboratories, Hercules, CA, USA) at room temperature. Derivative spectra and AUC values in the 340–700 nm region were obtained and analyzed using OriginPro (OriginLab Corporation, Northampton, MA, USA).

Encapsulation efficiency EE% was calculated as follows:

(5) $$EE\%=\frac{AUC(emulsion)-AUC(supernatant)}{AUC(emulsion)}\times 100\%,$$

where AUC (emulsion) is the AUC of the emulsion’s 2nd derivative spectrum, AUC (supernatant) corresponds to the supernatant’s 2nd derivative spectrum.

### 4.7. Cell Culture

Human breast adenocarcinoma MCF-7 and colon adenocarcinoma HCT116 cell lines were obtained from the American Type Culture Collection (Manassas, VA, USA). Non-malignant fibroblasts immortalized with *h*TERT cDNA were a gift of E.Dashinibaev, Engelhardt Institute of Molecular Biology, Russian Academy of Sciences, Moscow, Russia. Cells were routinely propagated in Dulbecco’s modified Eagle’s medium supplemented with 2 mM *L*-glutamine, 100 U/mL penicillin, 100 µg/mL streptomycin (all from PanEco, Russia) and 10% fetal bovine serum (HyClone, Logan, UT, USA) at 37 °C, 5% CO_2_ in a humidified atmosphere (normoxia). Prolonged hypoxia was achieved by continuous culture of HCT116 cells in the incubator with O_2_ displaced with N_2_ (1% O_2_, 5% CO_2_, 37 °C) for 8 weeks. For acute hypoxia, cells were treated with 2 mM sodium sulfite [65]. Cells in the logarithmic phase of growth were used in the experiments.

### 4.8. Intracellular Accumulation and Distribution

The HCT116 cells in 6-well plates (Nunc, Denmark; 2 × 10^5^ cells in 3 mL medium) were treated with **3b-PFC-NE** (10 µM chlorin) for up to 24 h at 37 °C, 20.9% O_2_, 5% CO_2_. After the completion of exposure, cells were washed with saline. Cell-associated fluorescence of **3b-PFC-NE** was analyzed by flow cytometry on a BD FACSCanto II (BD Biosciences, San Jose, CA) in the APC channel. For each sample, 5000 events were collected.

For intracellular distribution studies, HCT116 cells were grown in 35 mm Petri dishes with a glass bottom (SPL, Pyeongtaek, Republic of Korea) until 50% confluence. Then, **3b**, **3b-PFC-NE** (final concentration 10 μM chlorin) or blank emulsion (equivalent volume) was added for 24 h. After the completion of incubation, cells were washed with saline and stained with the following probes (Invitrogen Corp., Carlsbad, CA, USA) according to manufacturer’s recommendations: LysoTracker™ green (1 µM, 30 min), MitoTracker™ Green (0.5 µM, 20 min), the nuclear dye Hoechst 33342 (0.1 µg/mL, 10 min). After staining, the cells were washed with saline and subjected to confocal laser scanning microscopy at the following wavelengths: **3b** (λ_ex_ = 405 nm/λ_em_ = 650–720 nm), LysoTracker™ Green (λ_ex_ = 476 nm/λ_em_ = 490–560 nm), MitoTracker™ Green (λ_ex_ = 476 nm/λ_em_ = 490–560 nm), Hoechst 33342 (λ_ex_ = 405 nm/λ_em_= 415–470 nm). The confocal laser scanning microscope Leica TCS SP 5 equipped with LAS AF software (Leica Microsystems GmbH, Wetzlar, Germany) was used for imaging.

### 4.9. Cytotoxicity Studies

Compounds **3a–c** were dissolved in DMF as 5 mM stock solutions. These preparations and the respective NE formulations were stored at 4 °C until the experiments. Cells were plated in 96-well plates (Nunc, 5·10^3^ cells in 190 µL medium per well) and incubated for 24 h at 37 °C, 5% CO_2_. DMF solutions of **3a–c** or respective NE formulations were added to final concentrations of 1.5–15 μM (chlorin). The disodium salt of chlorin e6 (Photolon, Belmedpreparaty, Minsk, Belarus) was used as a reference PS. Dark cytotoxicity was assessed after 48 h of exposure.

For phototoxicity studies, emulsions were added to the cells for 24 h. Then, monolayers were washed with saline and illuminated with a 660 nm laser (AFC Polironics, Moscow, Russia) at a fluence rate of 8.3 mW/cm^2^, total fluence of 45 J/cm^2^ in the normoxic or hypoxic incubator (see Section 4.7) and incubated for an additional 24 h under the same conditions as during photoexcitation.

After the completion of drug exposure in the dark or incubation after illumination, 0.5 mg/mL 3-(4,5-dimethylthiazol-2-yl)-2,5-diphenyltetrazolium bromide (MTT reagent, PanEco, Moscow, Russia) was added to each well. Cells were incubated for 2 h, the medium was removed and formazan was solubilized in a mixture of DMSO-95% ethanol (*v*/*v* 2:1). The optical density of formazan solutions was measured on a Multiscan FC plate spectrophotometer (Thermo Scientific, Waltham, MA, USA) at 571 nm. Cell viability was calculated as OD_treated_/OD_control_ × 100%. Three independent experiments were performed for each concentration. Standard deviations did not exceed 10%. IC_50_ values were calculated using an online tool ‘Quest Graph™ IC50 Calculator’ (AAT Bioquest, Inc., Pleasanton, CA, USA) [66].

### 4.10. Mechanisms of Cell Photodamage

The HCT116 cells were grown in 35 mm Petri dishes with a glass bottom (SPL) until 50% confluence. Then **3b**, **3b-PFC-NE**, Photolon (final concentration 10 μM chlorin) or blank emulsion (equivalent volume) was added for 24 h. After the completion of incubation, cells were washed with saline and stained with the following probes (Invitrogen Corp.): lipid peroxidation sensor BODIPY™ 581/591 C_11_ (2.5 µM, 30 min), the nuclear dye Hoechst 33342 (0.1 µg/mL, 10 min). After staining, cells were washed with saline and illuminated with a 405 nm laser. Detection by confocal laser scanning microscopy was performed at the following wavelengths: BODIPY™ 581/591 C_11_ (λ_ex_ = 476 nm/λ_em_ = 510–590 nm for the reduced form and λ_ex_= 476 nm/λ_em_ = 590–690 nm for the oxidized form), Hoechst 33342 (λ_ex_ = 405 nm/λ_em_ = 415–470 nm). 

To monitor the photoinduced cell death depending on oxygen content, HCT116 cells in 35 mm Petri dishes (50% confluence) were incubated for 72 h in a normoxic or hypoxic incubator, respectively. Compound **3b**, **3b-PFC-NE** (each at final concentration 10 μM chlorin), blank emulsion (equivalent volume) or Photolon (final concentration 10 μM chlorin e6) was added for 24 h in normoxia or hypoxia, respectively. After the completion of incubation, the medium was replaced with saline containing 0.1 µg/mL Hoechst 33342. Ten minutes later, cells were washed with saline, and 10 µg/mL PI was added. Photoexcitation was performed with a 405 nm laser. Nuclear signals (Hoechst 33342) were detected as above. PI signals (λ_ex_ = 488 nm/λ_em_= 610–730 nm) were recorded within 30 min post illumination. The confocal laser scanning microscope Leica TCS SP 5 equipped with LAS AF software (Leica Microsystems GmbH, Germany) was used for imaging.

### 4.11. Statistics

Statistical analysis was performed using one-way analysis of variance (ANOVA) in GraphPad Prism 7 software. The Dunnett’s test was used to determine statistical significance between the groups treated with blank emulsion vs. **3a-c-PFC-NE** treated samples. Values *p* < 0.05 were considered statistically significant.

## 5. Conclusions

The objective of this work is the preparation of PFC-NEs with a PS dissolved in the fluorous phase for photodamage of tumor cells in hypoxia. Our design of nanoformulations presumed the modification of the chlorin macrocycle with perfluoroalkyl fragments of various lengths to achieve solubility in PFD as a gas transport phase. These modifications retained the spectral characteristics typical for chlorins, such as light absorption in the long wavelength region and the ability to generate singlet oxygen as a result of energy transfer from the excited triplet state to molecular oxygen. The developed technology of FC-PFC-NE preparation by ultrasonic emulsification allowed us to obtain stable NEs that contained 1.3 times more oxygen than the aqueous media, thereby making the emulsions applicable for light activation in normoxia and hypoxia. Emulsions accumulated in HCT116 cells after 2 h of incubation; the plateau was reached within 12–24 h. No significant dark cytotoxicity was registered within 48 h (IC_50_ > 15 μM). In contrast, upon photoexcitation of cells loaded with submicromolar concentrations of PS-containing PFC-NEs, lipid peroxidation followed by lethal photodamage of the plasma membrane were detectable in normoxia as well as in hypoxia. PS-free PFC-NEs were without effect; fluorous chlorins alone (without NEs) were significantly weaker than the full nanocarrier. The non-fluorinated chlorin e6 trisodium salt (reference PS) was one order of magnitude less efficient in normoxia and virtually inactive upon photoexcitation in hypoxia. Thus, our new nanoformulations were similarly potent in eliminating normoxic and hypoxic cells via the mechanism of photonecrosis.

## Figures and Tables

**Table 1:** Parameters of FC-PFC-NEs.

Emulsion	Storage Temperature, °C	z-Average Hydrodynamic Diameter, nm			PDI		
		Day 1	Day 7	Day 30	Day 1	Day 7	Day 30
**3a**-PFC-NE	+4	199.3 ± 2.7	209.5 ± 3.1	213.0 ± 2.9	0.084 ± 0.03	0.057 ± 0.02	0.098 ± 0.02
	−20		204.9 ± 3.6	196.4 ± 1.7		0.062 ± 0.03	0.120 ± 0.05
**3b**-PFC-NE	+4	200.6 ± 6.1	208.7 ± 5.6	221.1 ± 1.4	0.096 ± 0.01	0.027 ± 0.02	0.060 ± 0.04
	−20		214.9 ± 4.8	192.9 ± 2.5		0.120 ± 0.03	0.089 ± 0.04
**3c**-PFC-NE	+4	209.5 ± 4.0	211.8 ± 4.4	216.2 ± 4.1	0.054 ± 0.02	0.1 ± 0.03	0.036 ± 0.04
	−20		205.4 ± 3.7	191.7 ± 2.7		0.079 ± 0.02	0.085 ± 0.04

Shown are mean ± SD (*n* = 3).

**Table 2:** Optical characteristics of **3a**–**c** in DMF and PFC-NE.

Compound	Solvent	Absorption Maxima, nm					λ_Em_,nm	Φ_F_	Encapsulation Efficiency, % *
		Soret Band	Q_y_(1,0)	Q_y_(0,0)	Q_x_(1,0)	Q_x_(0,0)			
**3a**	DMF	407	504	530	598	650	660	0.22	-
	Emulsion	404	504	530	595	650	657	0.09	68.2 ± 3.2
**3b**	DMF	407	503	530	597	651	660	0.23	-
	Emulsion	402	504	530	596	650	658	0.10	61.5 ± 2.2
**3c**	DMF	407	503	530	597	651	660	0.23	-
	Emulsion	402	505	531	596	650	657	0.09	51.6 ± 7.8

* Mean ± SD (*n* = 3).

**Table 3:** Kinetic spectral characteristics of **3a**–**c** in DMF.

Compound	λ_max t-t_, nm	*k_T_* × 10^3^, s^−1^	*k*_q_ × 10^9^, M^−1^ × s^−1^	Φ_Δ_
**3a**	440	1.1	1.0	0.53
**3b**	440	1.1	1.1	0.48
**3c**	440	1.3	1.1	0.51

**Table 4:** Photoinduced cytotoxicity (IC_50_, μM) in human cell lines.

Compound	HCT116			MCF7		
	Normoxia	Cultured in Normoxia (20.9% O_2_), Photoexcitation in Hypoxia (1% O_2_)	Cultured in Hypoxia (1% O_2_), Photoexcitation in Normoxia (20.9% O_2_)	Normoxia	Cultured in Normoxia (20.9% O_2_), Photoexcitation in Hypoxia (1% O_2_)	Cultured in Hypoxia (1% O_2_), Photoexcitation in Normoxia (20.9% O_2_)
**3a**-PFC-NE	1.0 ± 0.2	3.8 ± 0.2 ***	1.1 ± 0.1	0.4 ± 0.04	2.3 ± 0.2 ***	0.3 ± 0.03
**3b**-PFC-NE	1.7 ± 0.1	4.4 ± 0.1 ***	1.4 ± 0.1	0.5 ± 0.04	4.3 ± 0.3 ***	0.5 ± 0.03
**3c**-PFC-NE	2.2 ± 0.2	6.2 ± 0.1 ***	2.3 ± 0.1	0.6 ± 0.03	5.8 ± 0.5 ***	0.6 ± 0.05
**3a**-DMF	>15	>15	>15	>15	>15	>15
**3b**-DMF	>15	>15	>15	13.6 ± 1.2	>15	>15
**3c**-DMF	>15	>15	>15	11.0 ± 0.9	>15	>15
Photolon	9.2 ± 0.9	>15	9.5 ± 0.9	8.1 ± 0.8	>15	8.8 ± 0.7

Values are mean ± SE of three measurements. *** *p* < 0.001 compared to blank emulsion.

## Data Availability

Not applicable.

## Supplementary Materials

The following supporting information can be downloaded at: https://www.mdpi.com/article/10.3390/ijms24097995/s1.
