# Supplementary material for: Perfluorocarbon Nanoemulsions with Fluorous Chlorin-Type Photosensitizers for Antitumor Photodynamic Therapy in Hypoxia
Source: Int J Mol Sci. 2023 Apr 28;24(9):7995. doi: 10.3390/ijms24097995 (PMC10178184; doi:10.3390/ijms24097995)

Supplementary materials

Perfluorocarbon Nanoemulsions with Fluorous Chlorin Type Photosensitizers for Antitumor Photodynamic Therapy in Hypoxia

Minh Tuan Nguyen[1*], Elizaveta V. Guseva[2], Aida N. Ataeva[3], Andrey L. Sigan[2], Anna V. Shibaeva[1], Maria V. Dmitrieva[4], Ivan D. Burtsev[1], Yulia L. Volodina[4], Alexandra S. Radchenko[1], Anton E. Egorov[1], Alexey A. Kostyukov[1], Pavel V. Melnikov[5], Nikolai D. Chkanikov[2], Vladimir A. Kuzmin[1], Alexander A. Shtil[4,6], Alina A. Markova [1,2*]

[1] Emanuel Institute of Biochemical Physics, Russian Academy of Sciences, 4 Kosygin Street, 119334 Moscow, Russian Federation
[2] A.N. Nesmeyanov Institute of Organoelement Compounds, Russian Academy of Sciences, 28 Vavilov Street, 119991 Moscow, Russian Federation
[3] Pirogov Russian National Research Medical University, 1 Ostrovitianov Street, 117997 Moscow, Russian Federation
[4] Blokhin National Medical Research Center of Oncology, 24 Kashirskoe shosse, 115522, Moscow, Russian Federation
[5] M.V. Lomonosov Institute of Fine Chemical Technologies, MIREA—Russian Technological University, 86 Vernadsky Avenue, 119571 Moscow, Russian Federation
[6] Institute of Cyber Intelligence Systems, National Research Nuclear University MEPhI, 31 Kashirskoe shosse, 115409, Moscow, Russian Federation
* Correspondence: tuantonyx@yahoo.com; alenmark25@gmail.com

1. General synthetic procedure

Equipment: NMR spectra ^1H and ^{19}F were recorded on a Bruker AMX-400 and AMX-300 spectrometer with frequency 400.13 and 376.50 MHz at 20°C. Chemical shifts (δ) were determined relatively to proton signal of solvent ($CDCl_3$) for ^1H and trifluoroacetic acid (TFA) as external standard for ^{19}F.

The ESI (1) and APCI (2) mass-spectra were registered on the Finnigan LCQ Advantage tandem dynamic mass-spectrometer. Nitrogen 10/0 (1) or 70/10 (2) served as a sheath and auxiliary gas. Flow rate of acetonitrile: 50 µl/min (1) or 350 µl/min (2). The temperature of the heated capillary was 150°C, the electric potential between the needle and the counter electrode was 4.5 kV (1) or 6.0 kV (2). Samples with the solution 10^{-4} mol/l in acetonitrile were introduced into the ion source through the Reodyne injector with 5 µl loop. Mass spectra EI-DIP were recorded on a Finnigan Polaris Q, ionization energy 70 eV, procedure of sample injection: DIP. High resolution mass spectra (HRMS) were recorded on the Maxismass spectrometer (Bruker Daltonic, Germany). Mass spectra MALDIwere recorded on a MALDI-TOF Shimadzu AXIMA Confidence. A DHB (2,5-dihydroxybenzoic acid) matrix was used for sample preparation. Ions were recorded using a reflectron detector in the high resolution mode.

Monitoring of the reaction and purity of products was controlled using Merck Kieselgel 60 F254 plates. For column chromatography, the silica gel (MN Kieselgel 60) and aluminum oxide (neutral, 100-200 µm) were used. Elemental analysis was performed in the Laboratory of elemental analysis at the A.N. Nesmeyanov Institute of Organoelement Compounds, Russian Academy of Sciences.

Reagents and solvents: acetonitrile, chloroform ($CHCl_3$), ethyl acetate and toluene were dried using standard procedures. Pentane, hexane, methanol and Freon-113 were used without additional purifications. Pyrrole and triethylamine (Et_3N) were distilled before use. Polyfluorinated alcohols and pentafluorobenzaldehyde were purchased from "P&M Invest". Other reagents were purchased from Acros Organics (Moscow, Russia) and ABCR GmbH (Karlsruhe, Germany) and were used without purification.

Synthesis

Synthesis of aldehydes **1a-c**. General procedure.

Pentafluorobenzaldehyde (1 g, 5.1 mmol), acetonitrile (10 ml) were placed in a 100 ml round-bottom flask, Et_3N (0.85 ml, 6.1 mmol) was added dropwise with stirring - the solution turned slightly yellow. Then the corresponding polyfluoroaliphatic alcohol was added (5.2 mmol). The reaction mixture was refluxed for 4-5 h; the reaction was monitored by TLC. After cooling the reaction mixture the solvent was removed on a rotary evaporator. The residue was distributed between hexane (10 ml) and water (10 ml). $NaHCO_3$ was added until gas formation ceased. The system separated into three phases from top to bottom: slightly colored hexane phase - aqueous solution - yellow-brown oil. The hexane phase was separated. The product was extracted with hexane 4-5 times until the oil phase disappeared (a small amount of black resin may remain). The combined hexane phases were dried over Na_2SO_4. The solution was filtered and solvent was removed on a rotary evaporator. Residue was dried in vacuo (1 mbar) at room temperature. This method of isolation provides the product purity sufficient for the next reaction step. To obtain products with high purity, aldehydes **1a** and **1b** were distilled in vacuo; **1c** was crystallized from pentane.

*4-(1,1-H,H-perfluoropropyl-1-oxy)-2,3,5,6-tetrafluorobenzaldehyde (**1a**):* Light-yellow oil, yield 83%.NMR ^{1}H(CDCl$_3$, δ, ppm, *J*/Hz): 4.78 (t, 2H, *J*=11.98, OC<u>H</u>$_2$), 10.25 (s, 1H, C<u>H</u>O). ^{19}F NMR (CDCl$_3$, δ, ppm, *J*/Hz): -79.47 (m, 2F, C_{Ar}-F^{orto}), -68.50 (m, 2F, C_{Ar}-F^{meta}), -48.16 (s, 2F, C<u>F</u>$_2$), -7.13 (s, 3F, C<u>F</u>$_3$). MS (EI-DIP), m/z: 325 [M-H]$^+$, 193 [M-$CH_2C_2F_5$]$^+$, 69 [CF_3]$^+$.

*4-(1,1-H,H-perfluoropenthyl-1-oxy)-2,3,5,6-tetrafluorobenzaldehyde (**1b**):* Light-yellow oil, yield 86%, b.p. 105°C/2mbar. NMR ^{1}H (CDCl$_3$, δ, ppm, *J*/Hz): 4,83 (t, 2 H, OC<u>H</u>$_2$CF$_2$, *J* = 12.72); 10.26 (s, 1 H, C<u>H</u>O). ^{19}F NMR (CDCl$_3$, δ, ppm, *J*/Hz): -78.18 (m, 2 F, C_{Ar}-F^{ortho}); -67.30 (m, 2 F, C_{Ar}-F^{meta}); -48.77 (s, 2 F, C<u>F</u>$_2$); -46.53 (s, 2 F, C<u>F</u>$_2$); -43.28 (s, 2 F, C<u>F</u>$_2$); -3.37 (s, 3 F, C<u>F</u>$_3$). MS (EI-DIP), m/z: 425 [M-H]$^+$, 193 [M-$CH_2C_4F_9$]$^+$, 151 [CF_3CFHCF_2]$^+$, 69 [CF_3]$^+$.

*4-(1,1-H,H-perfluorohepthyl-1-oxy)-2,3,5,6- tetrafluorobenzaldehyde (**1c**):* Light-yellow powder, yield 89%. NMR ^{1}H (CDCl$_3$, δ, ppm, *J*/Hz): 4,82 (t, 2 H, OC<u>H</u>$_2$CF$_2$, *J* = 12.55); 10.27 (s, 1 H, C<u>H</u>O). ^{19}F NMR (CDCl$_3$, δ, ppm, *J*/Hz): -78.08 (m, 2 F, C_{Ar}-F^{ortho}); -67.15 (m, 2 F, C_{Ar}-F^{meta}); -48.54 (s, 2 F, C<u>F</u>$_2$); -45.51 (s, 2 F, C<u>F</u>$_2$); -45.19 (s, 2 F, C<u>F</u>$_2$); -44.53 (s, 2 F, C<u>F</u>$_2$); -42.98 (s, 2 F, C<u>F</u>$_2$); -3.18 (s, 3 F, C<u>F</u>$_3$). MS (EI-DIP), m/z: 525 [M-H]$^+$, 193 [M-$CH_2C_6F_{13}$]$^+$, 69 [CF_3]$^+$.

Synthesis of porphyrins **2a-c**. General procedure.

Aldehydes **1a-c** (2.3 mmol) and chloroform (75 mL) were placed into a 100 mL round-bottom flask with stirring. Pyrrole (0.17 g, 2.5 mmol) was added dropwise, I_2 (0.12 g, 0.46 mmol) was added. The reaction mixture was refluxed for 4 h and cooled. Chloranil (1.13 g, 4.6 mmol) was added, and the mixture was refluxed for 2 h. After cooling the solvent was removed on a rotary evaporator. The residue was placed in a Soxhlet apparatus and the product was extracted with freon 113. The resulting solution was evaporated on a rotary evaporator. The residue (1.27 g) was put onto a layer of silica gel (10 x 2 cm) and washed off with a mixture of chloroform - hexane (1:1). The resulting solution was dried over Na_2SO_4, the solvent was removed on a rotary evaporator. The residue was put in methanol (7 ml), boiled, cooled to room temperature and left overnight. The precipitate was filtered off, washed with a small amount of cold methanol, and dried on air.

*meso-tetrakis(4-(1,1-H,H-perfluoropentyl-1-oxy)-2,3,5,6-tetrafluorophenyl)porphyrin (**2a**):* Purple powder, yield 23%.NMR ^{1}H(CDCl$_3$, δ, ppm, *J*/Hz): -2.89 (s, 2H, N<u>H</u>); 5.02 (t, 8H, *J* = 12.08), 8.96 (s, 8H, Pyr-<u>H</u>). ^{19}F NMR (CDCl$_3$, δ, ppm, *J*/Hz): -78,55 (m, 8F, C_{Ar}-F^{orto}); -59.44 (m, 8F, C_{Ar}-F^{meta}); -46.55 (s, 8F, C<u>F</u>$_2$); -5,52 (s, 12F, C<u>F</u>$_3$).MS (MALDI), m/z: 1495.22 [M+H]$^+$.

*meso-tetrakis(4-(1,1-H,H-perfluoropentyl-1-oxy)-2,3,5,6-tetrafluorophenyl)porphyrin (**2b**):* Purple powder, yield 28%, m.p. > 300°C. NMR ^{1}H(CDCl$_3$, δ, ppm, *J*/Hz): -2.89 (s, 2H, N<u>H</u>); 5.07 (t, 8H, *J* = 12.87), 8.94 (s, 8H, Pyr-<u>H</u>). ^{19}F NMR (CDCl$_3$, δ, ppm, *J*/Hz): -78.51 (m, 8F,

C_{Ar}-F^{orto}); -59.44 (m, 8F, C_{Ar}-F^{meta}); -48.45 (s, 8F, C<u>F</u>$_2$), -46.20 (s, 8F, C<u>F</u>$_2$); -42.93 (s, 8F, C<u>F</u>$_2$), -3.04 (s, 12F, C<u>F</u>$_3$). MS (APCI), m/z: 1895.4 [M+H]$^+$.

<u>*meso-tetrakis(4-(1,1-H,H-perfluoropentyl-1-oxy)-2,3,5,6-tetrafluorophenyl)porphyrin* (***2c***):</u> Purple powder, yield 25%. NMR^{1}H(CDCl$_3$, δ, ppm, *J*/Hz): -2.86 (s, 2H, N<u>H</u>), 5.11 (t, 8H, J_1= 12.79, J_2= 12.56, OC<u>H</u>$_2$), 8.97 (s, 8H, Pyr H). ^{19}F NMR (CDCl$_3$, δ, ppm, *J*/Hz): -78.48 (m, 8F, C_{Ar}-F^{ortho}), -59.44 (dd, 8F, J_1= 13.79, J_2= 8.19, C_{Ar}-F^{meta}), -48.38 (m, 8F, CF$_2$), -45.14 (m, 8F, CF$_2$), -44.33 (m, 8F, CF$_2$), -42.73 (m, 8F, CF$_2$), -2.98 (t, 12F, *J* = 9.05, CF$_2$). Anal. calcd. for$C_{72}H_{18}F_{68}N_4O_4$ (%): C, 35.00; H, 0.43; N, 2.00; F, 56.50.Found (%): C, 37.68; H, 0.79; N, 2.44; F, 56.30

<u>Synthesis of chlorins **3a-c**. General procedure.</u>

To a solution of porphyrins **2a-c** (0.10 g, 1 eq.) in toluene (25 ml) N-methylglycine (2 eq.) and paraformaldehyde (4.7 eq.) were added. The mixture was refluxed for 5 h under argon atmosphere. The reaction was monitored by TLC. Depending on the reactivity of substrates, additional portions of N-methylglycine (2 eq.) and paraformaldehyde (4.7 eq.) were added, and the mixture was refluxed for another 5 h. Then the mixture was evaporated under reduced pressure. The residue was purified by column chromatography on silica gel with gradient elution (chloroform - ethyl acetate).

<u>*2,3,3a,21a-tetrahydro-2-methyl-5,10,15,20-tetrakis[2,3,5,6-tetrafluoro-4-(1H,1H-perfluoropropyl-1-oxy)phenyl]-1H,23H,25H-pyrrolo[3,4-β]porphin* (***3a***):</u> Dark purple powder with green shimmer, yield 30%. NMR ^{1}H(CDCl$_3$, δ, ppm, *J*/Hz): -1.86 (s, 2H, N<u>H</u>), 2.74 (s, 3H, C<u>H</u>$_3$N), 3.06 (s, 2H, C<u>H</u>$_2$N), 4.19 (s, 2H, C<u>H</u>$_2$N), 4.95-5.09 (m, 8H, C<u>H</u>$_2$CF$_2$), 5.78 (s, 2H, C<u>H</u>), 8.38 (m, 2H, Pyr-H), 8.55 (s, 2H, Pyr-H), 8.78 (m, 2H, Pyr-H). ^{19}F NMR (CDCl$_3$, δ, ppm, *J*/Hz): -80.45 - -80.18 (m, 4F), -79.06 (m, 2F), -77.30 (m, 2F), -62.05 (m, 2F), -61.70 (m, 4F), -60.99 (m, 2F), -48.55 (m, 8F), -7.53 (m, 12F). MS (MALDI), m/z: 1552.67 [M+H]$^+$.

<u>*2,3,3a,21a-tetrahydro-2-methyl-5,10,15,20-tetrakis[2,3,5,6-tetrafluoro-4-(1H,1H-perfluoropentyl-1-oxy)phenyl]-1H,23H,25H-pyrrolo[3,4-β]porphin* (***3b***):</u> Dark purple powder with green shimmer, yield 65%. NMR ^{1}H(CDCl$_3$, δ, ppm, *J*/Hz): -1.93 (s, 2H, N<u>H</u>), 2.73 (s, 3H, C<u>H</u>$_3$N), 3.08 (s, 2H, C<u>H</u>$_2$N), 4.19 (s, 2H, C<u>H</u>$_2$N), 5.00-5.13 (m, 8H, C<u>H</u>$_2$CF$_2$), 5.78 (s, 2H, C<u>H</u>), 8.38 (m, 2H, Pyr-H), 8.55 (s, 2H, Pyr-H), 8.79 (m, 2H, Pyr-H). ^{19}F NMR (CDCl$_3$, δ, ppm, *J*/Hz): -80.45, -80.17, -79.06, -77.28, -62.13, -61.74, -60.98, -50.49, -48.25, -45.00, -5.08. MS (MALDI), m/z: 1952.78 [M+H]$^+$.

<u>*2,3,3a,21a-tetrahydro-2-methyl-5,10,15,20-tetrakis[2,3,5,6-tetrafluoro-4-(1H,1H-perfluoroheptyl-1-oxy)phenyl]-1H,23H,25H-pyrrolo[3,4-β]porphin* (***3c***):</u> Dark purple powder with green shimmer, yield 39%. NMR ^{1}H(CDCl$_3$, δ, ppm, *J*/Hz): -1.93 (s, 2H, N<u>H</u>), 2.70 (s, 3H, C<u>H</u>$_3$N), 3.05 (s, 2H, C<u>H</u>$_2$N), 4.18 (m, 2H, C<u>H</u>$_2$N), 4.98-5.12 (m, 8H, C<u>H</u>$_2$CF$_2$), 5.75 (s, 2H, C<u>H</u>), 8.37 (m, 2H, Pyr-H), 8.55 (s, 2H, Pyr-H), 8.78 (m, 2H, Pyr-H). ^{19}FNMR (CDCl$_3$, δ, ppm, *J*/Hz): -80.36, -79.10, -77.35, -62.11, -61.77, -61.07, -50.41, -47.26, -47.05, -46.34, -44.76, -42.72, -5.03. HRMS, m/z: calcd. for $C_{75}H_{26}F_{68}N_5O_4$ [M+H]$^+$ 2352.0893, found 2352.0886.

2. Theoretical calculations of oxygen content in PFC

At 25°C and 1 atm O_2, the volume fractions of oxygen in PFD and water are 51% and 3.2%, respectively (https://detector-cooling.web.cern.ch/data/Fluoro_Solubility.htm). Then the theoretical value of oxygen content in a 2% (v/v) PFC-NE under the same conditions is 0.02*51+0.98*3.2 = 4.156%, which is 4.156%/3.2% = 1.3 times higher than in water.

3. Spectroscopic studies of FC in DMF and PFC-NE

To obtain absorption spectra of FC in PFC-NE, we removed background absorbance from light scattering. The simplest method is to use the emulsion without FC as a blank; however the difference in the nanodroplet sizes between blank and FC-PFC can lead to under- or over-correction of absorbance. For this reason, we used a mathematical model for baseline correction (Figure S1).

Absorbance is defined as:

$$A_{sc} = \lg\left(\frac{I_0}{I}\right),$$

where A_{sc} is absorbance from light scattering, I_0 is the intensity of incident light, I is the intensity of transmitted light.

It is known that the ratio between I/I_0 is proportional to $1/\lambda^4$ in Rayleigh scattering, which can be applied to particles smaller than 1/10th wavelength. However, the diameter of PFC nanodroplets is ~200 nm, which is larger than 1/10th wavelength of UV/Vis spectrum. Therefore, the power will be different from 4, and we will denote it as n.

$$\frac{I}{I_0} \sim \frac{1}{\lambda^n},$$

Thus, we can assume that $\lg(A_{sc})$ is proportional to $n\lg(\lambda)$:

$$10^{A_{sc}} = \frac{I_0}{I},$$

$$\frac{I_0}{I} \sim \lambda^n,$$

$$10^{A_{sc}} \sim \lambda^n,$$

$$lgA_{sc} \sim n \times lg\lambda,$$

To test this assumption, we recorded the absorption spectrum of blank PFC-NE without FC and found a linear dependence of $\lg(A_{sc})$ on $n\lg(\lambda)$:

$$lgA_{sc} = -n \times lg\lambda + b,$$

where $A_{sc} = A_{exp} - A_{solvent}$ (without zero-ing)

Three absorption minimums in absorption spectra of **3a-c** in DMF(571, 623 and 680 nm) presume that absorbance in these wavelengths in PFC-NE spectrum originates predominantly from background scattering. One can assume the equations:

$$\begin{cases} \lg A_{min1} = -n \times \lg \lambda_{min1} + b \\ \lg A_{min2} = -n \times \lg \lambda_{min2} + b \\ \lg A_{min3} = -n \times \lg \lambda_{min3} + b \\ \lg A_{scx} = -n \times \lg \lambda_{scx} + b \end{cases},$$

where A_{min1}, A_{min2}, A_{min3} are absorption minima; λ_{min1},λ_{min2},λ_{min3} are wavelength at absorption minima; A_{scx} is absorbance from the light scattering at wavelength x (λ_{scx}).

Solving the system of equations we find absorbance of background light scattering at wavelength x:

$$A_{scx} = 10^{\left(\lg(A_{571} \times A_{623} \times A_{680}) - \frac{\lg\left(\frac{A_{623} \times A_{680}}{A_{571}{}^2}\right) \times \lg\left(\frac{571 \times 623 \times 680}{x^3}\right)}{\lg\left(\frac{623 \times 680}{571^2}\right)}\right) \times \frac{1}{3}},$$

and calculate absorbance of FC in PFC-NE at wavelength x without background:

$$A_x = A_{exp} - A_{solvent} - A_{scx},$$

where A_x is the background-corrected absorbance of FC at wavelength x.

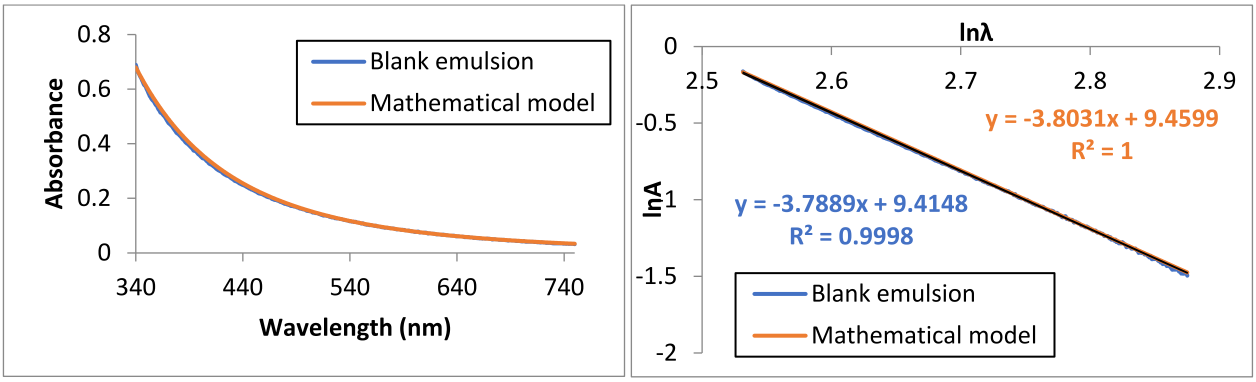

Figure S1. Absorption spectra of the emulsion.

Left, absorption spectrum of the blank emulsion. *Right*, linear regression of experimental and calculated values.

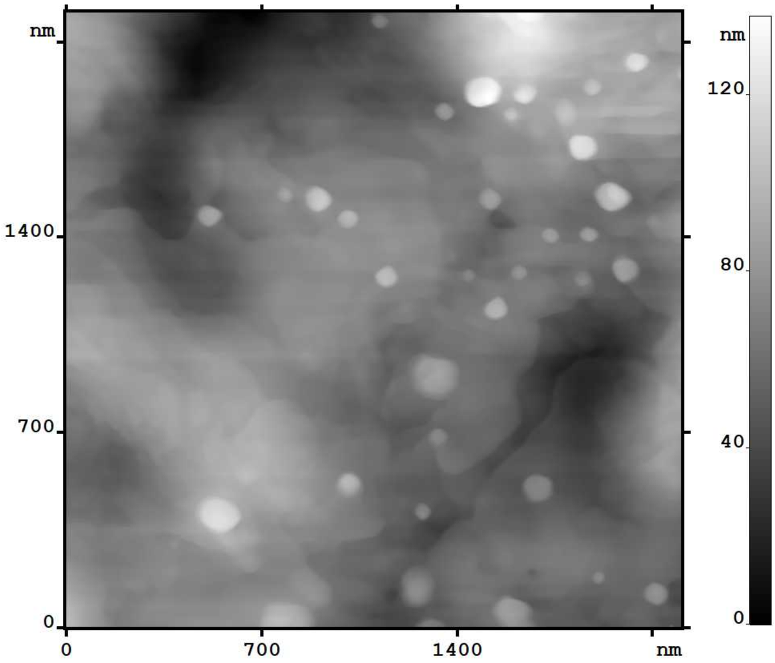

Figure S2. Atomic force microscopy images of **3b-PFC-NE** particles.

White, spherical particles 90±20 nm in diameter. The Nanoscope V Multimode (Bruker, Billerica, MA), high resolution high resonant frequency polysilicon AFM Cantilevers HA_FM (Estonia) with silicon tips with typical resonant frequency 77 kHz (dispersion ±10%) were used for analysis.

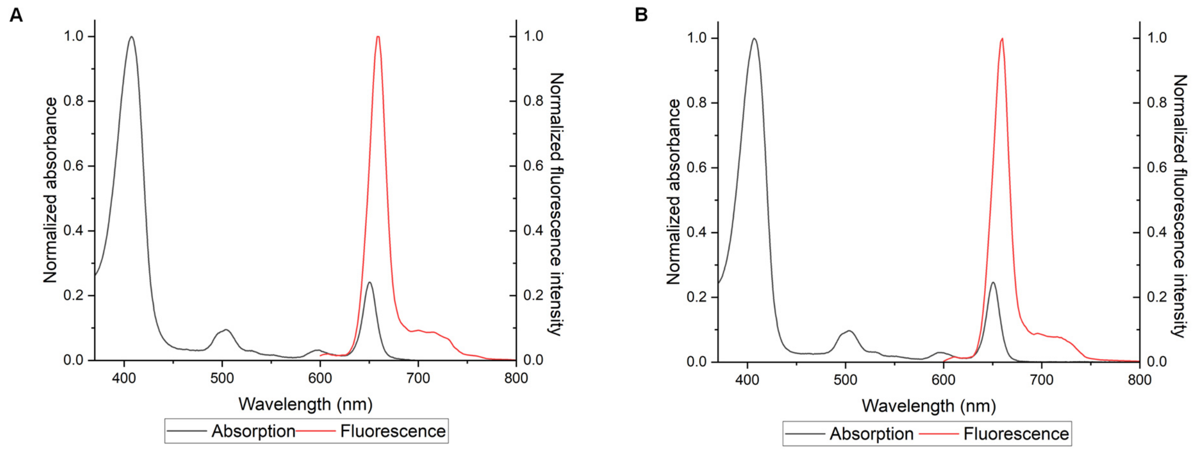

Figure S3. Absorption and fluorescence spectra of A) **3a** and B) **3c** in DMF.

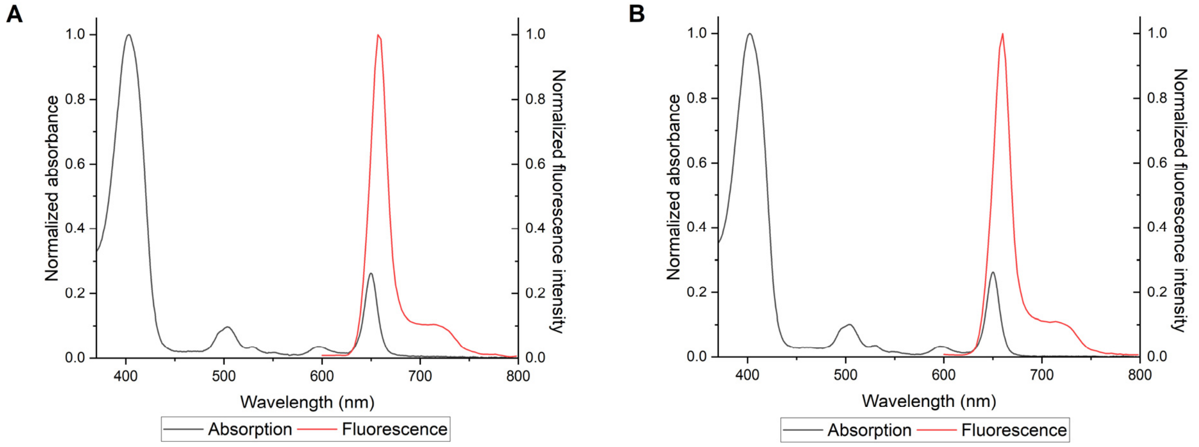

Figure S4. Absorption and fluorescence spectra of A) **3a** and B) **3c** in PFC-NE.

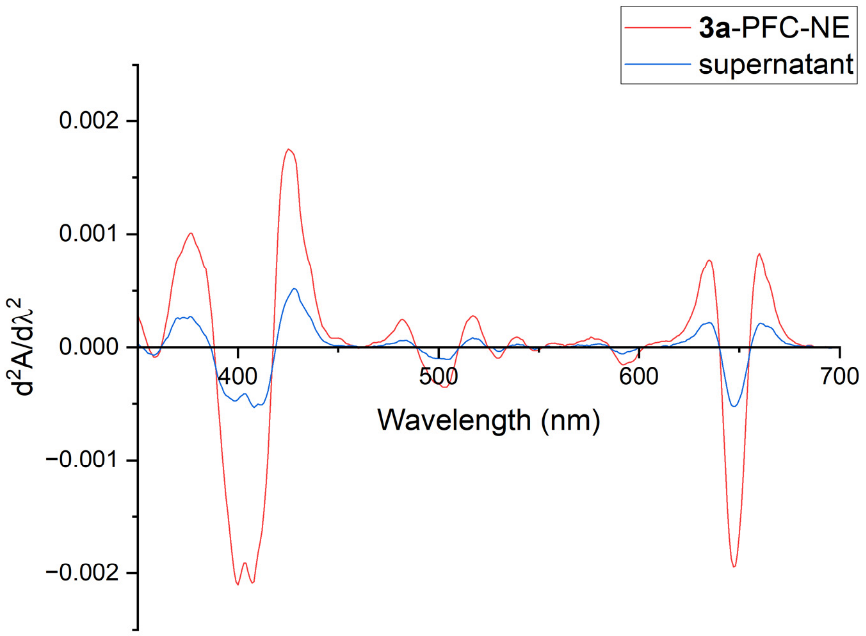

Figure S5. 2nd derivative spectra of **3a**-PFC-NE and its supernatant.

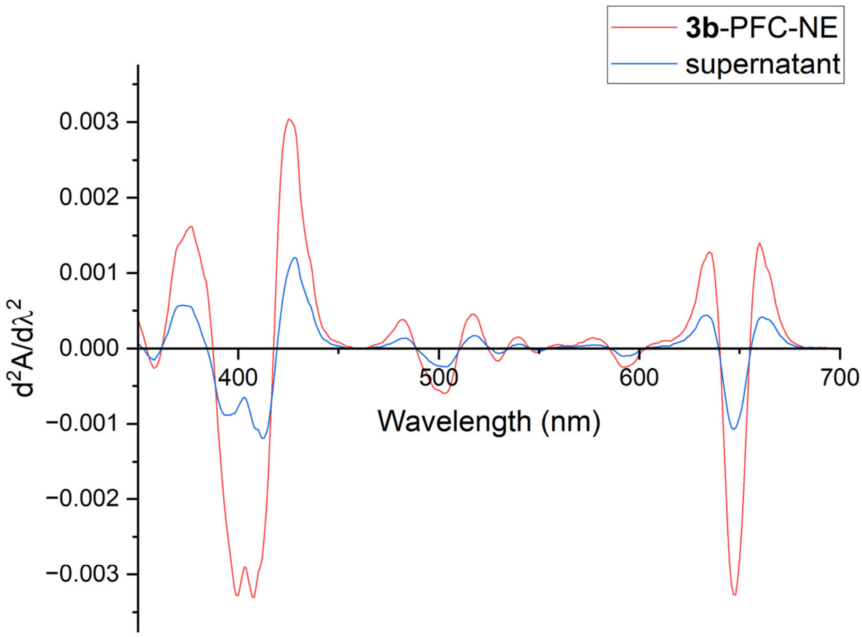

Figure S6. 2nd derivative spectra of **3b**-PFC-NE and its supernatant.

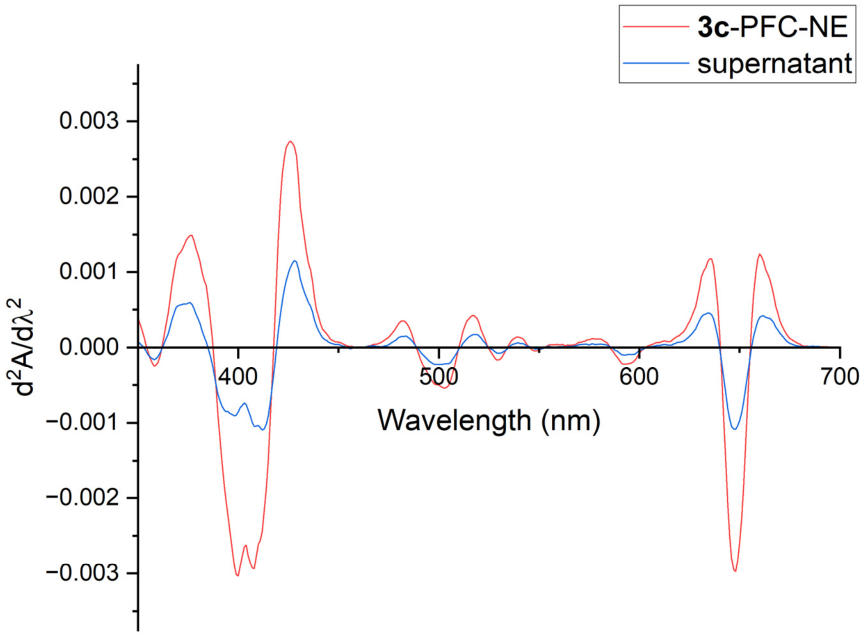

Figure S7. 2nd derivative spectra of **3c**-PFC-NE and its supernatant.

Table S1. Encapsulation efficiency analysis.

Compound		EE% using $d^2A/d\lambda^2$ at 650 nm	EE% using AUC of whole spectra
**3a**	Emulsion	69.3±3.4	68.2±3.2
	Supernatant		
**3b**	Emulsion	65.4±1.7	61.5±2.2
	Supernatant		
**3c**	Emulsion	54.0±8.2	51.6±7.8
	Supernatant		

Shown are mean±SD (n=3).

4. Triplet states of FC

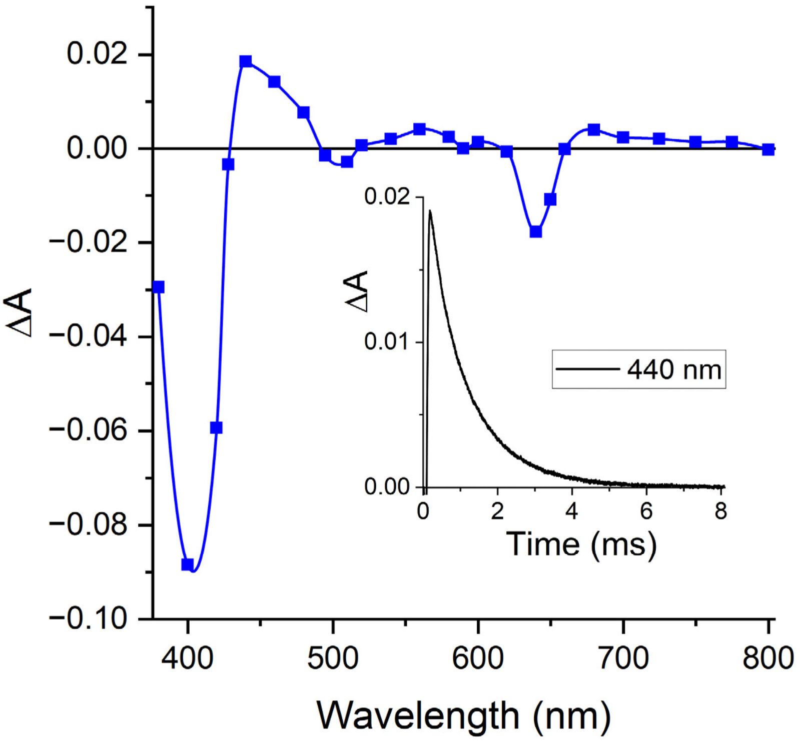

Figure S8. Transient absorption spectrum of **3a** (4.5×10^{-7} M) in DMF (300 μs after flash). Inset: kinetics of the triplet state decay.

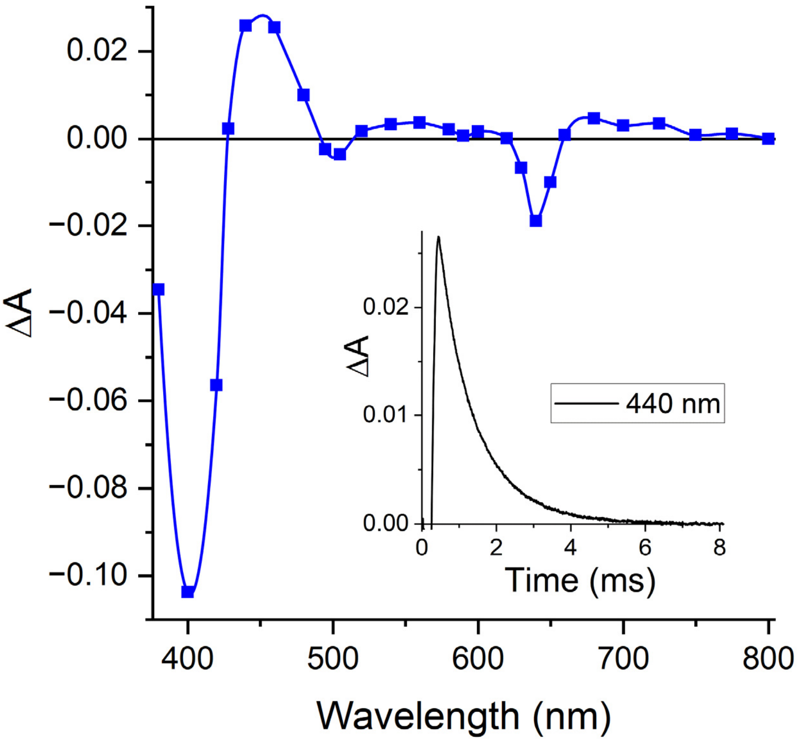

Figure S9. Transient absorption spectrum of **3c** (3.7×10^{-7} M) in DMF (300 μs after flash). Inset: kinetics triplet state decay kinetics.

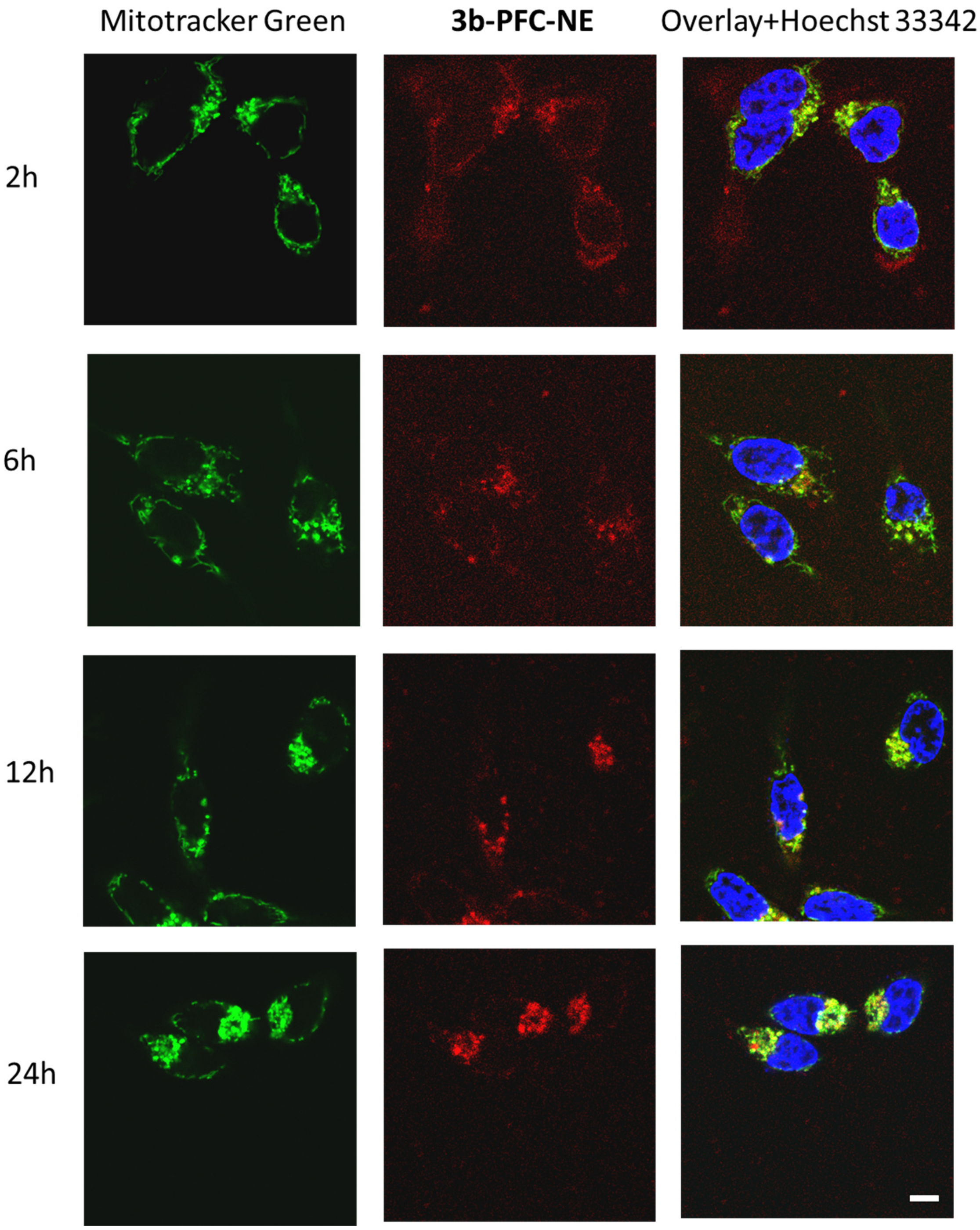

Figure S10. Intracellular distribution of 3b-PFC-NE (10 μM chlorin). Fluorescence signals: green – MitoTracker Green, red – **3b**, blue – Hoechst 33342. Bar, 5 μm.

The HCT116 cells were loaded with **3b-PFC-NE** for 2-24 h, washed, stained with intracellular dyes and analyzed by confocal laser scanning microscopy. By 2 h **3b-PFC-NE** was detectable at the cell periphery as diffuse inclusions; by 6 h the emulsion distributed across the cytoplasm and was partially colocalized with mitochondria. By 12-24 h the mitochondrial localization was more pronounced.

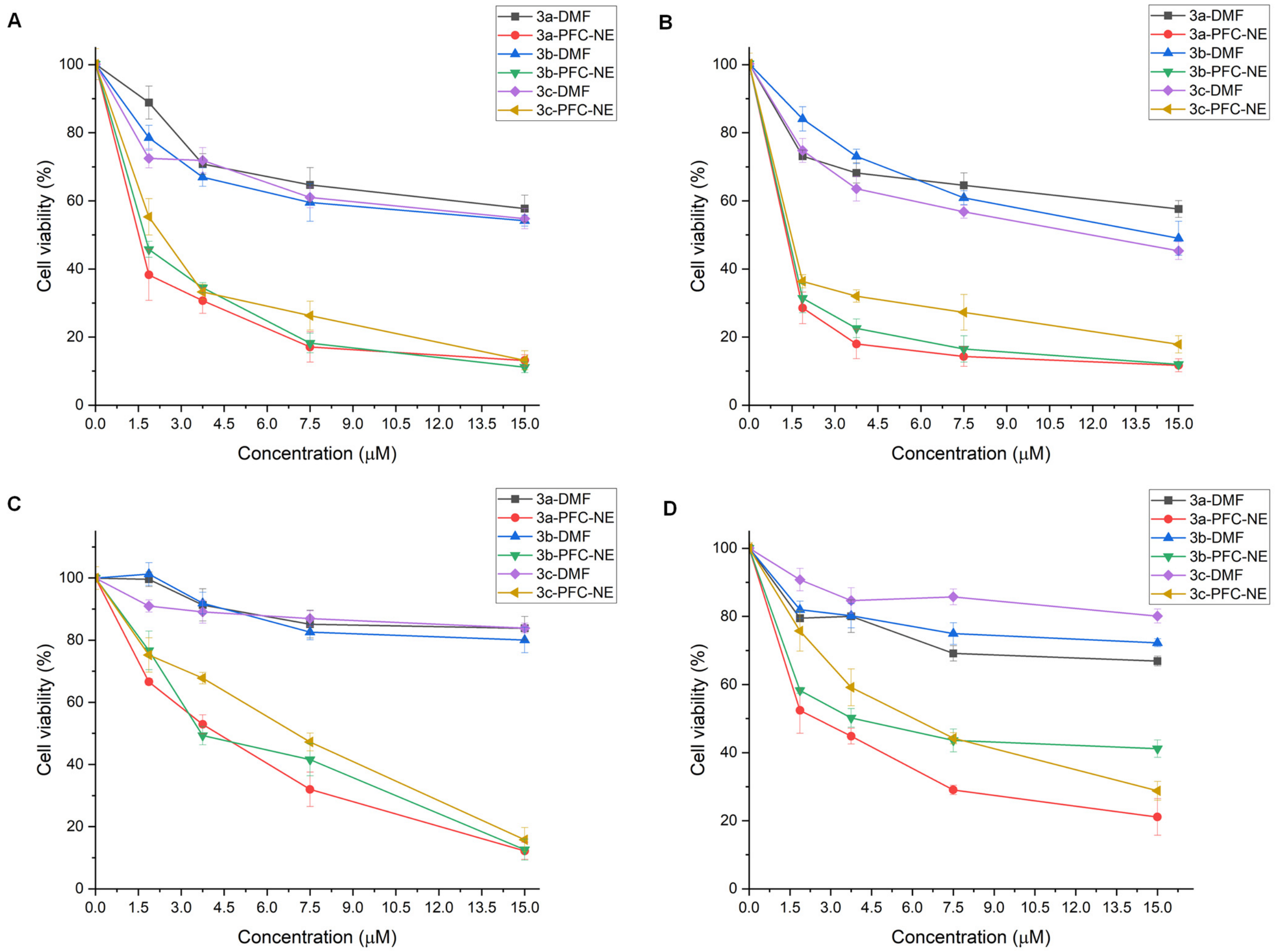

Figure S11. Photocytotoxicity of FC-PFC-NE (660 nm, 8.3 mW/cm^2, 45 J/cm^2).

Normoxic conditions: A) HCT116 cells, B) MCF-7 cells. Hypoxic conditions: C) HCT116 cells, D) MCF-7 cells. MTT were performed 24 h post illumination. Values are mean ± SE of 3 measurements with <10% error.

Table S2. Photoinduced cytotoxicity (IC_{50}, μM) in non-malignant fibroblasts.

Compound	Non-malignant fibroblasts
**3a-PFC-NE**	9.2±0.8
**3b-PFC-NE**	8.3±0.8
**3c-PFC-NE**	11.9±0.9
Photolon	13.5±1.0

Values are mean ± SE of 3 measurements with <10% error.

^{1}H- and ^{19}F-NMR spectra of synthesized compounds

*4-(1,1-H,H-perfluoropropyl-1-oxy)-2,3,5,6-tetrafluorobenzaldehyde (**1a**)*

NMR 1H

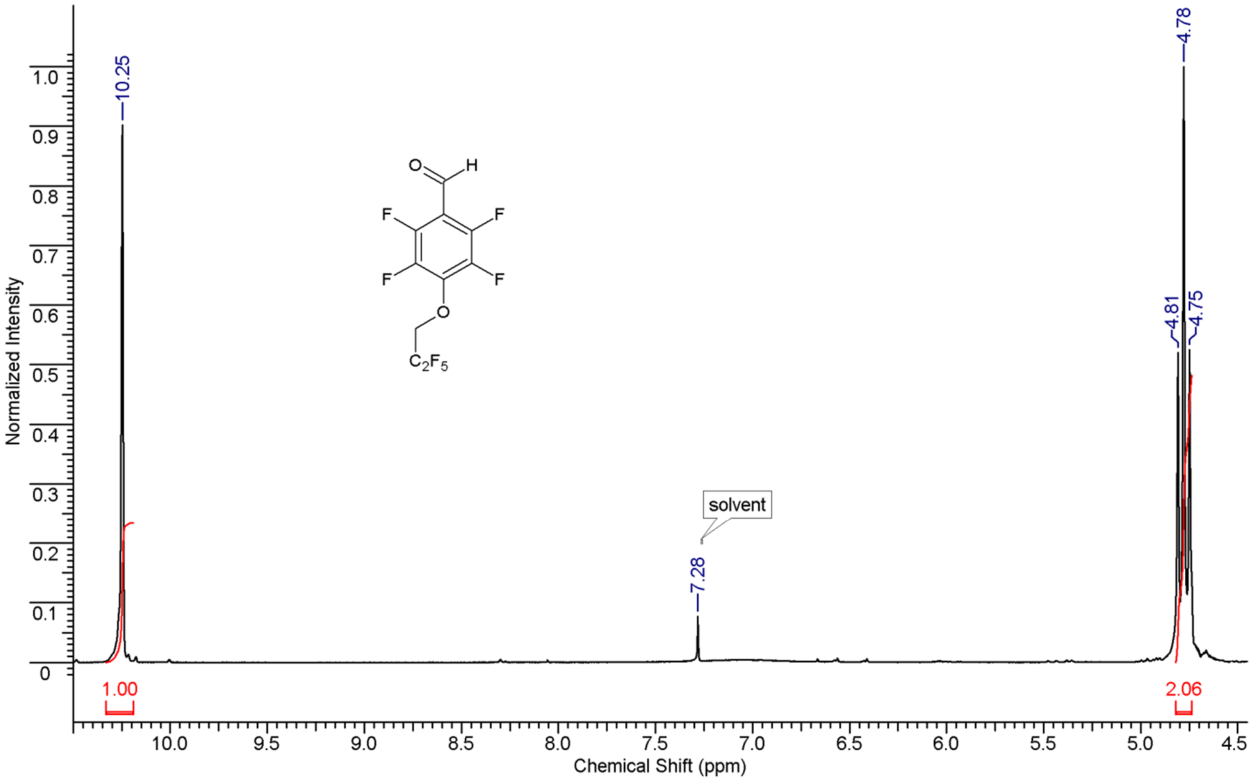

NMR 19F (external standard $CFCl_3$)

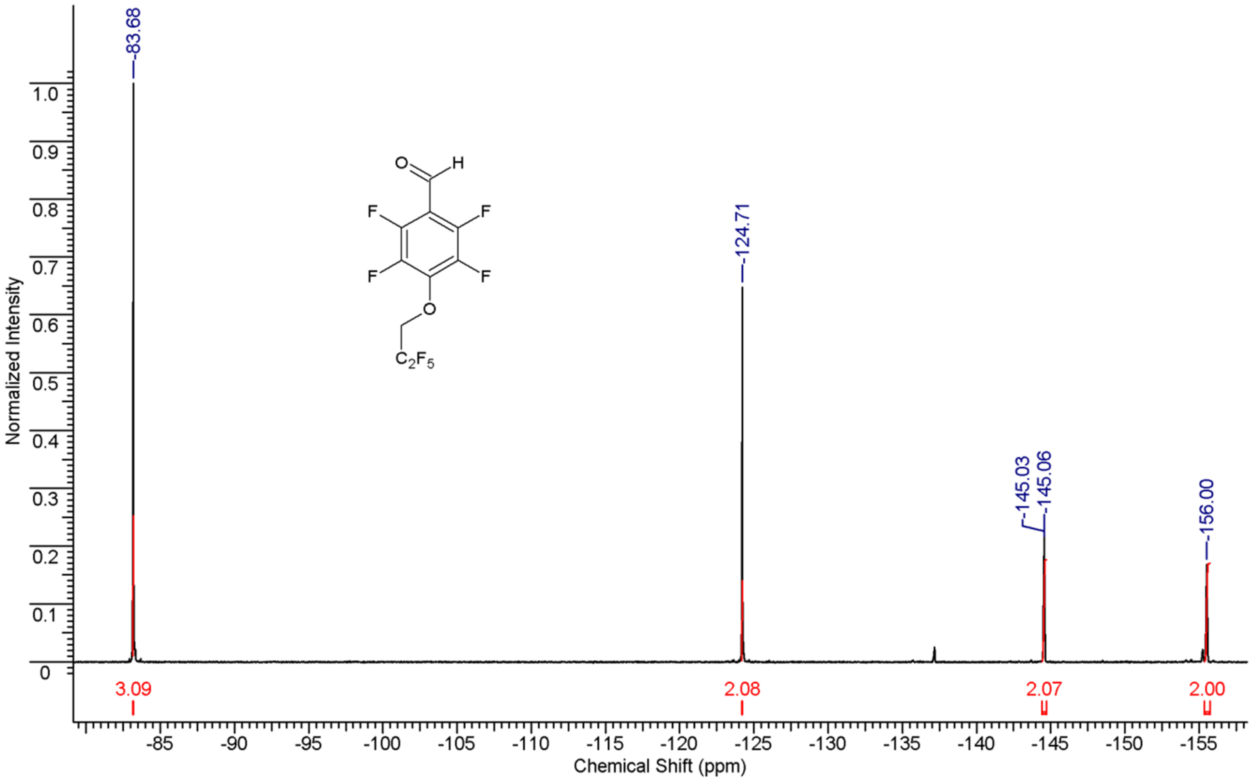

*4-(1,1-H,H-perfluoropenthyl-1-oxy)-2,3,5,6-tetrafluorobenzaldehyde (**1b**)*

NMR 1H

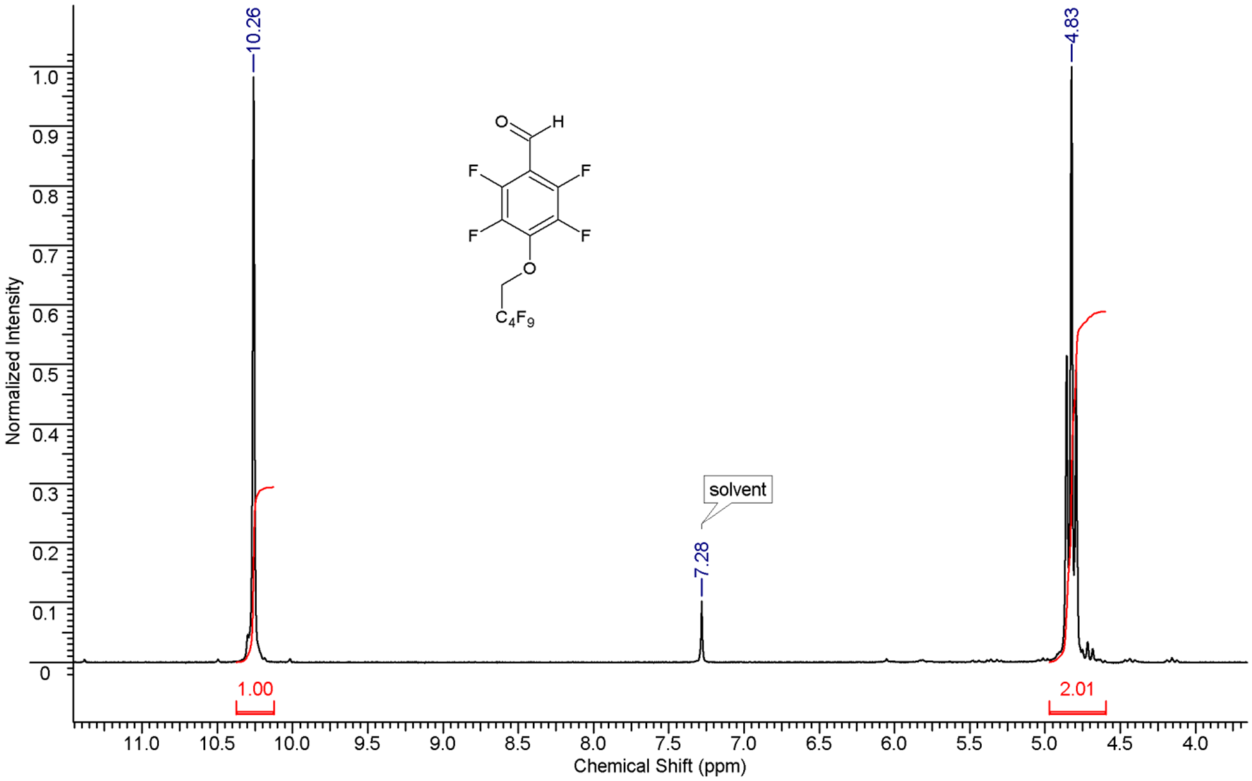

NMR 19F (external standard CF_3COOH)

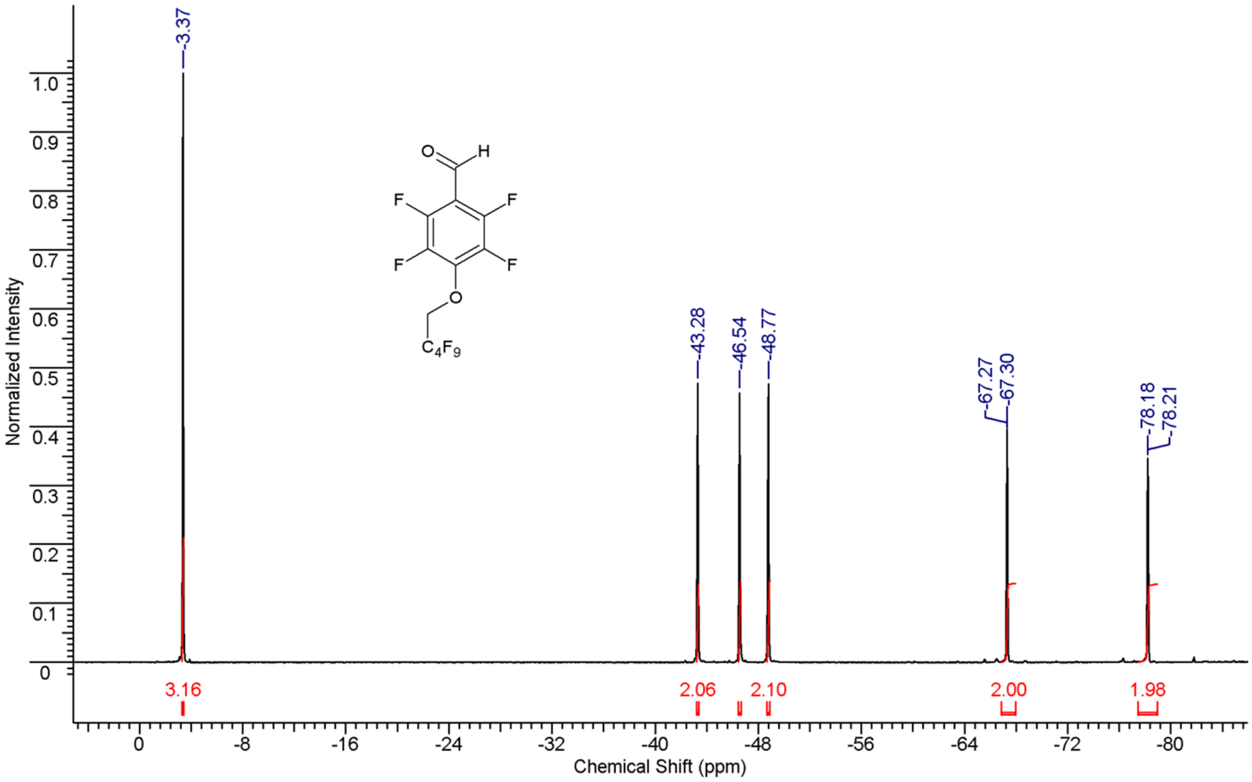

*4-(1,1-H,H-perfluorohepthyl-1-oxy)-2,3,5,6- tetrafluorobenzaldehyde (**1c**)*

NMR 1H

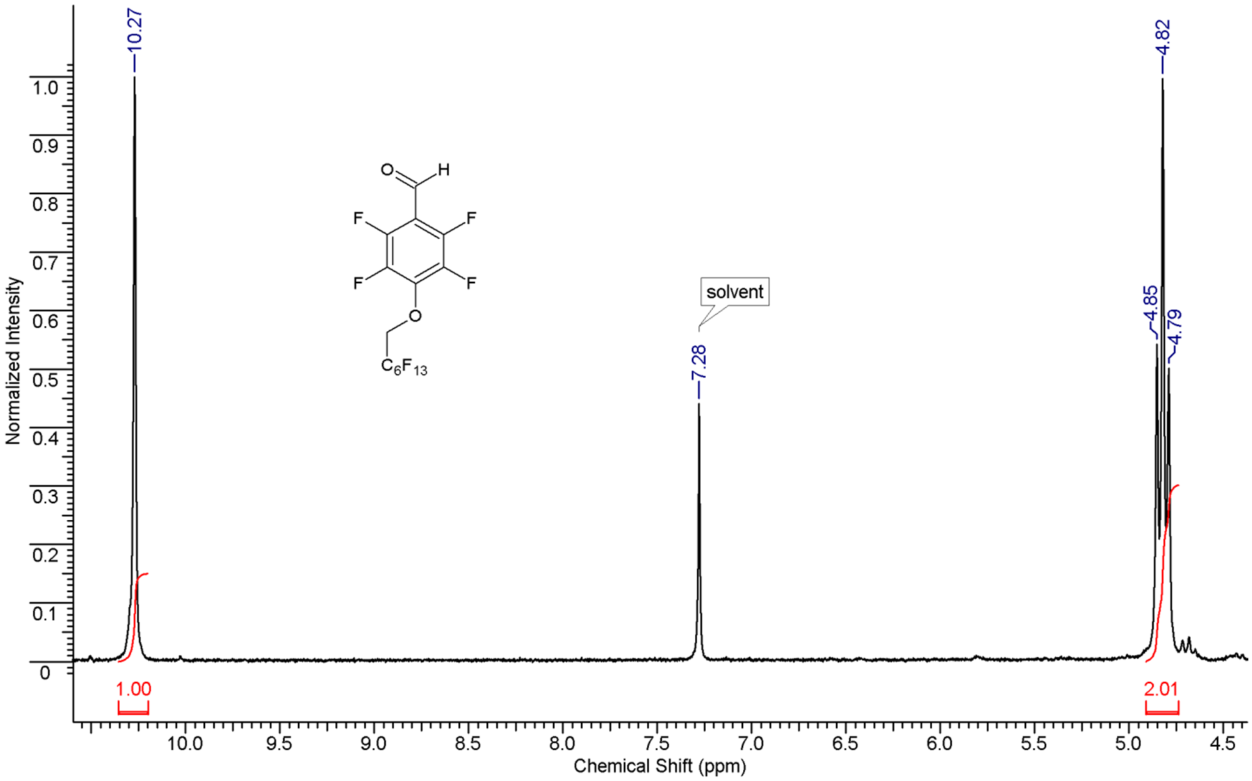

NMR 19F (external standard $CFCl_3$)

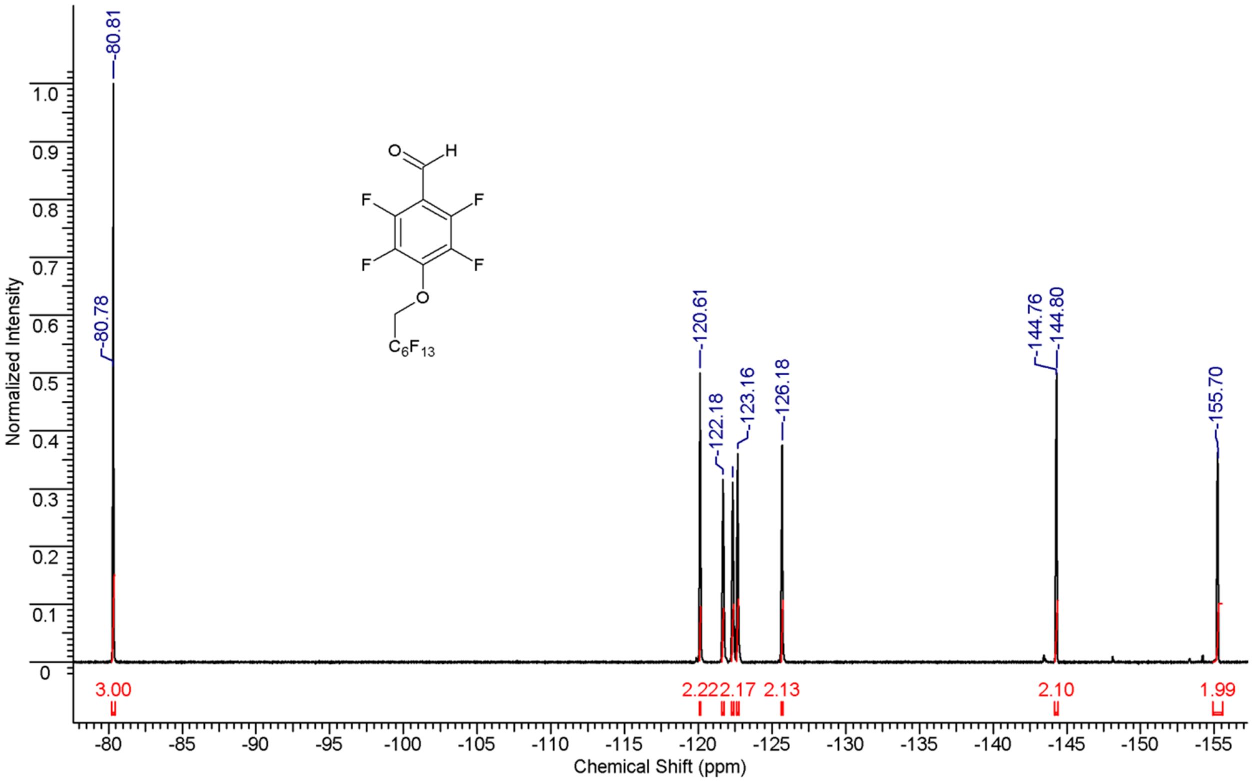

*meso-tetrakis(4-(1,1-H,H-perfluoropentyl-1-oxy)-2,3,5,6-tetrafluorophenyl)porphyrin (**2a**)*

NMR 1H

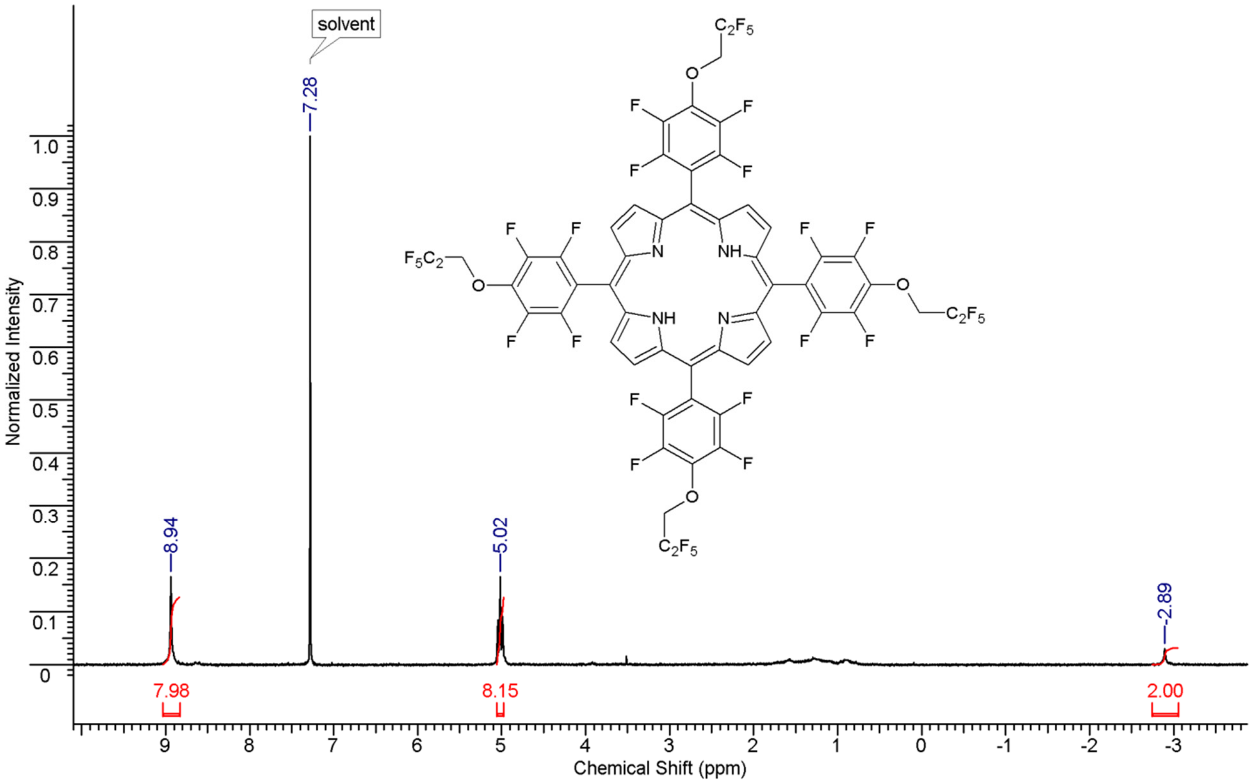

NMR 19F (external standard CF_3COOH)

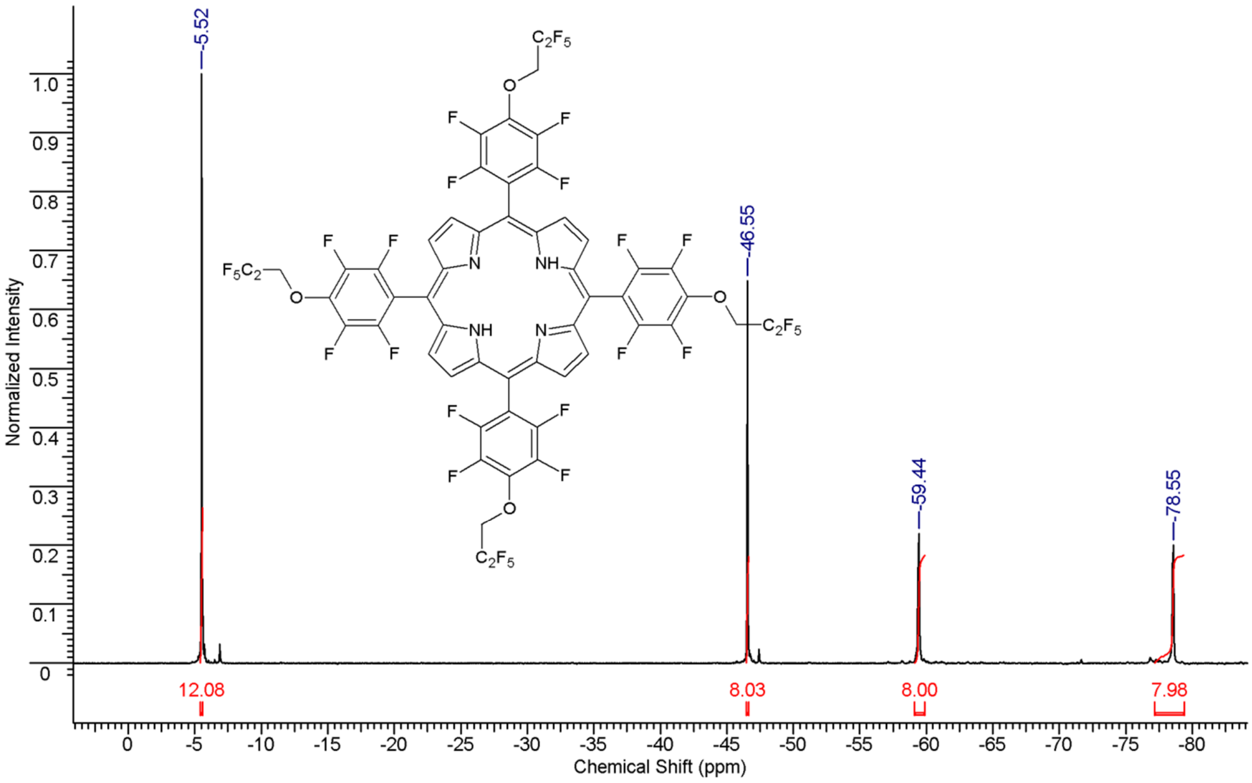

*meso-tetrakis(4-(1,1-H,H-perfluoropentyl-1-oxy)-2,3,5,6-tetrafluorophenyl)porphyrin (**2b**)*

NMR 1H

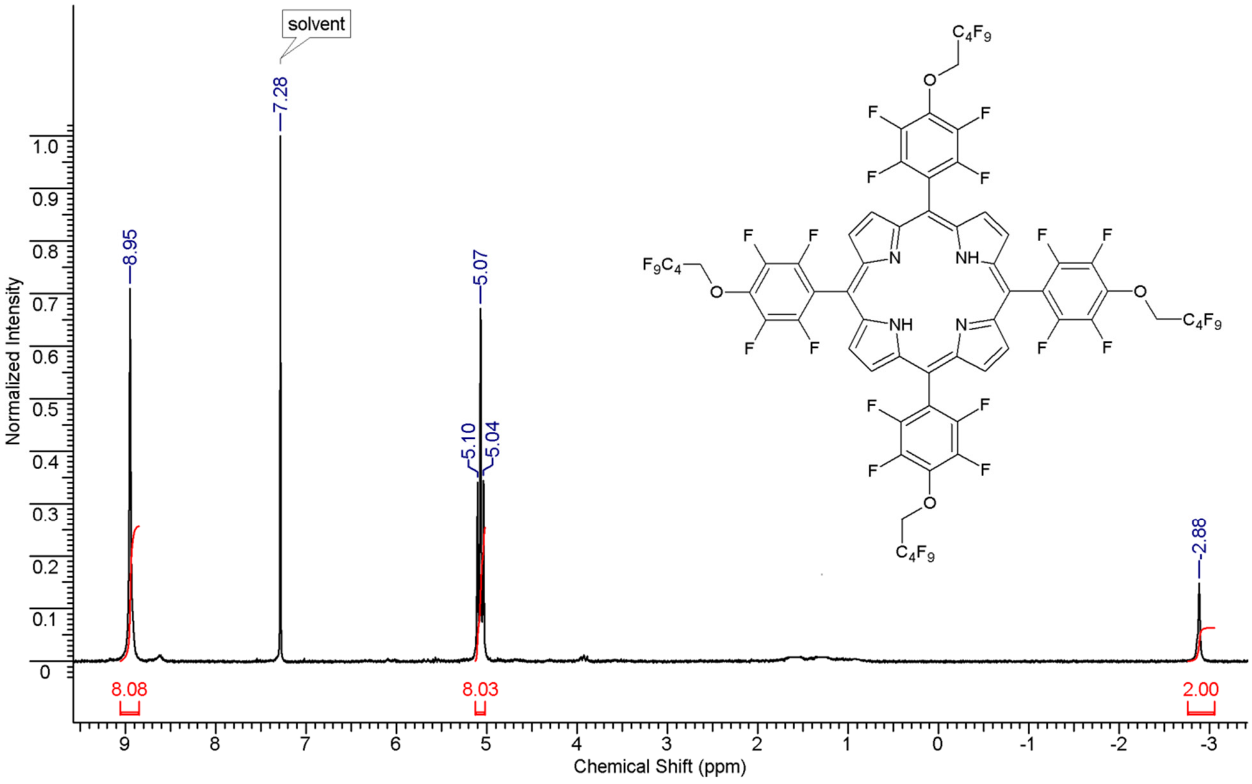

NMR 19F (external standard CFCl3)

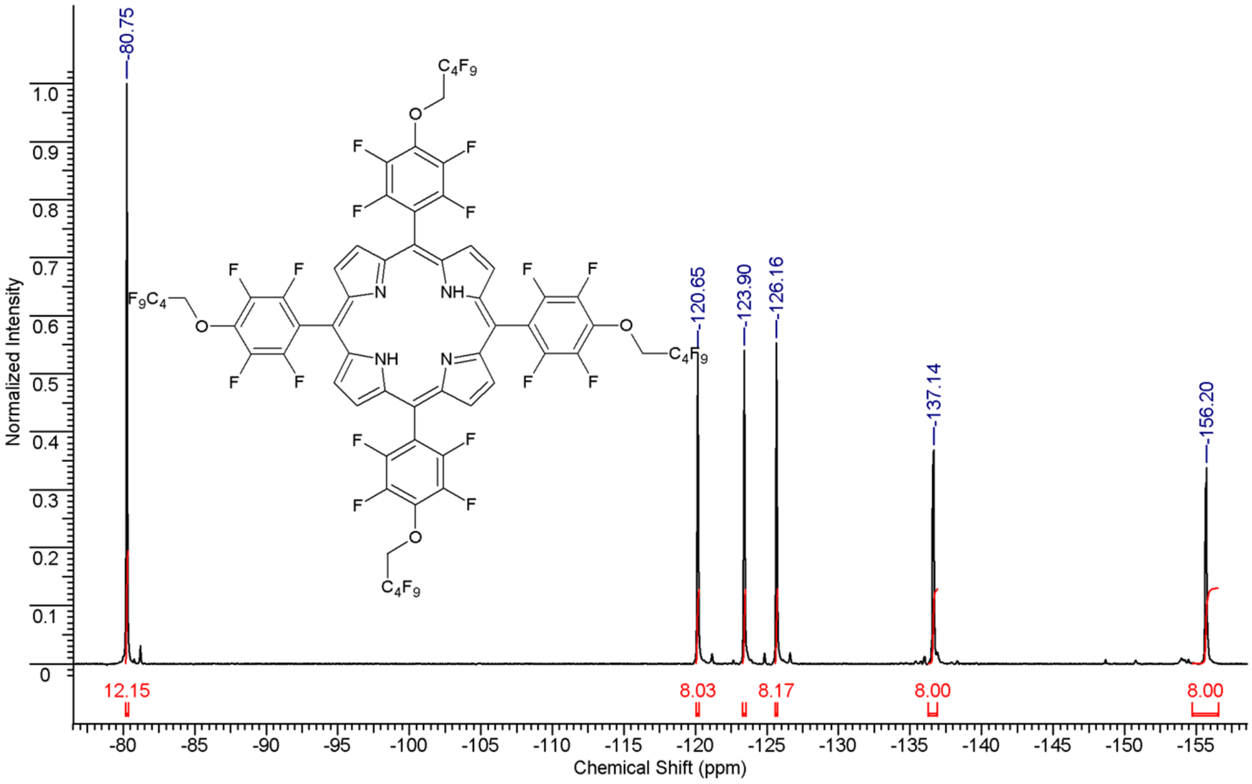

*meso-tetrakis(4-(1,1-H,H-perfluoropentyl-1-oxy)-2,3,5,6-tetrafluorophenyl)porphyrin (**2c**)*

NMR 1H

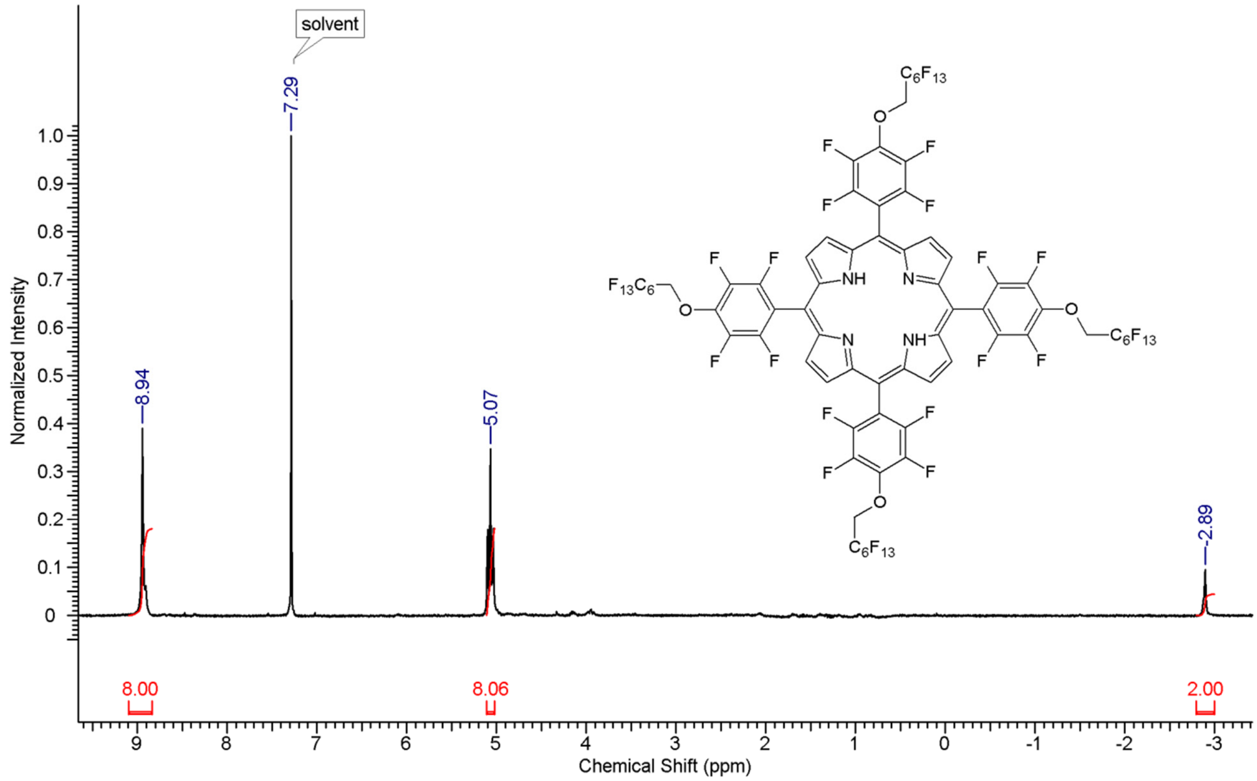

NMR 19F (external standard $CFCl_3$)

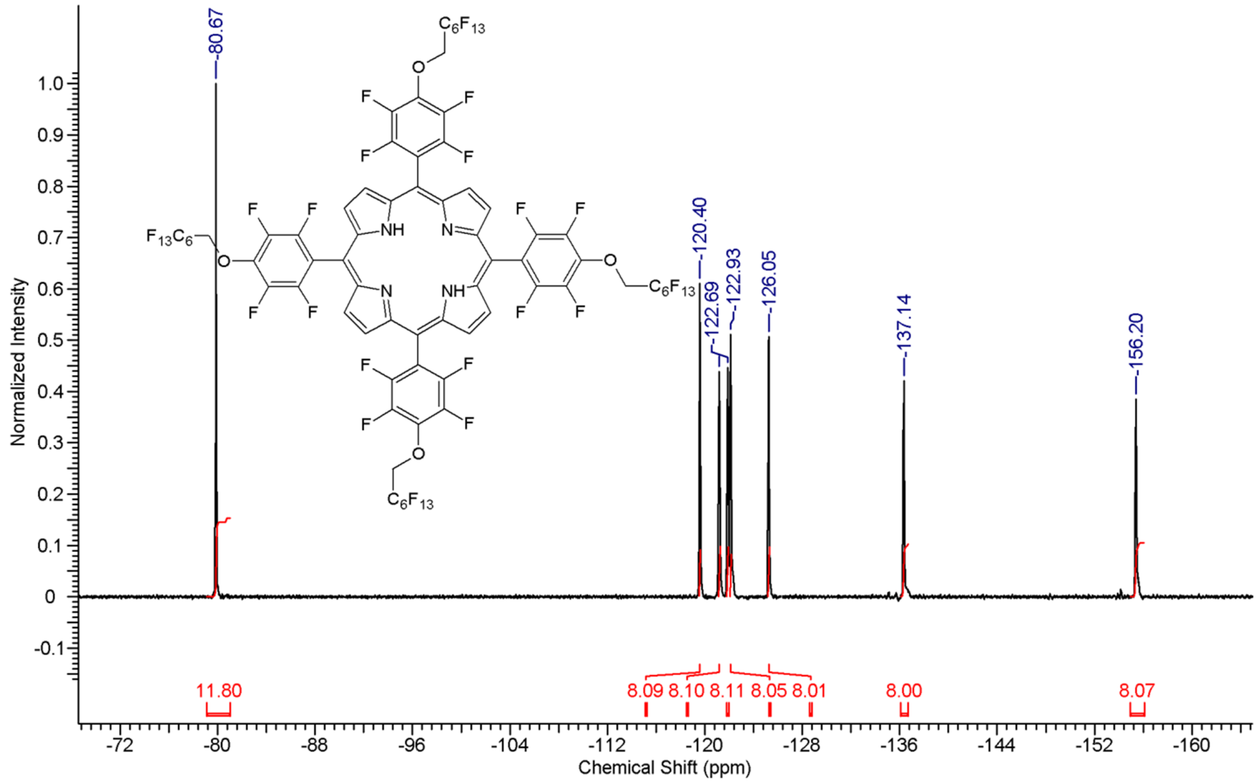

2,3,3a,21a-tetrahydro-2-methyl-5,10,15,20-tetrakis[2,3,5,6-tetrafluoro-4-(1H,1H-perfluoropropyl -1-oxy)phenyl]-1H,23H,25H-pyrrolo[3,4-β]porphin ***(3a)***

NMR 1H

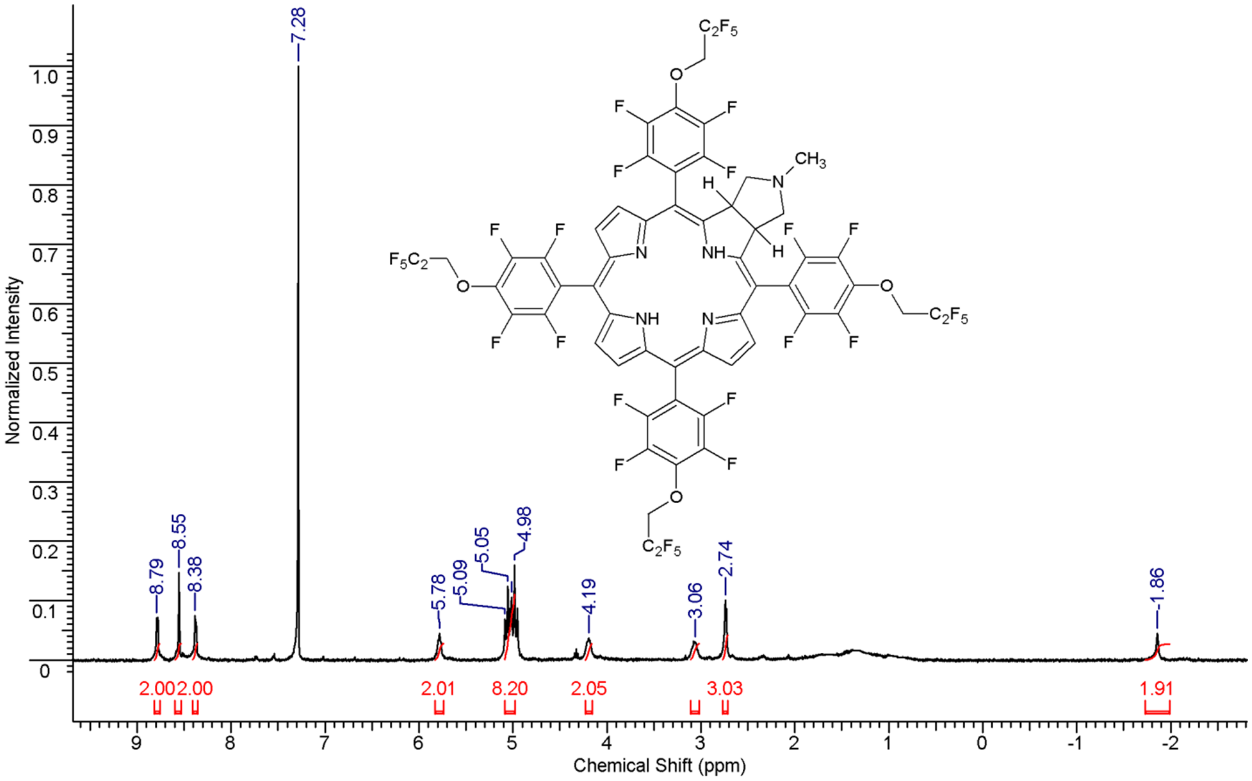

NMR 19F (external standard CFCl3)

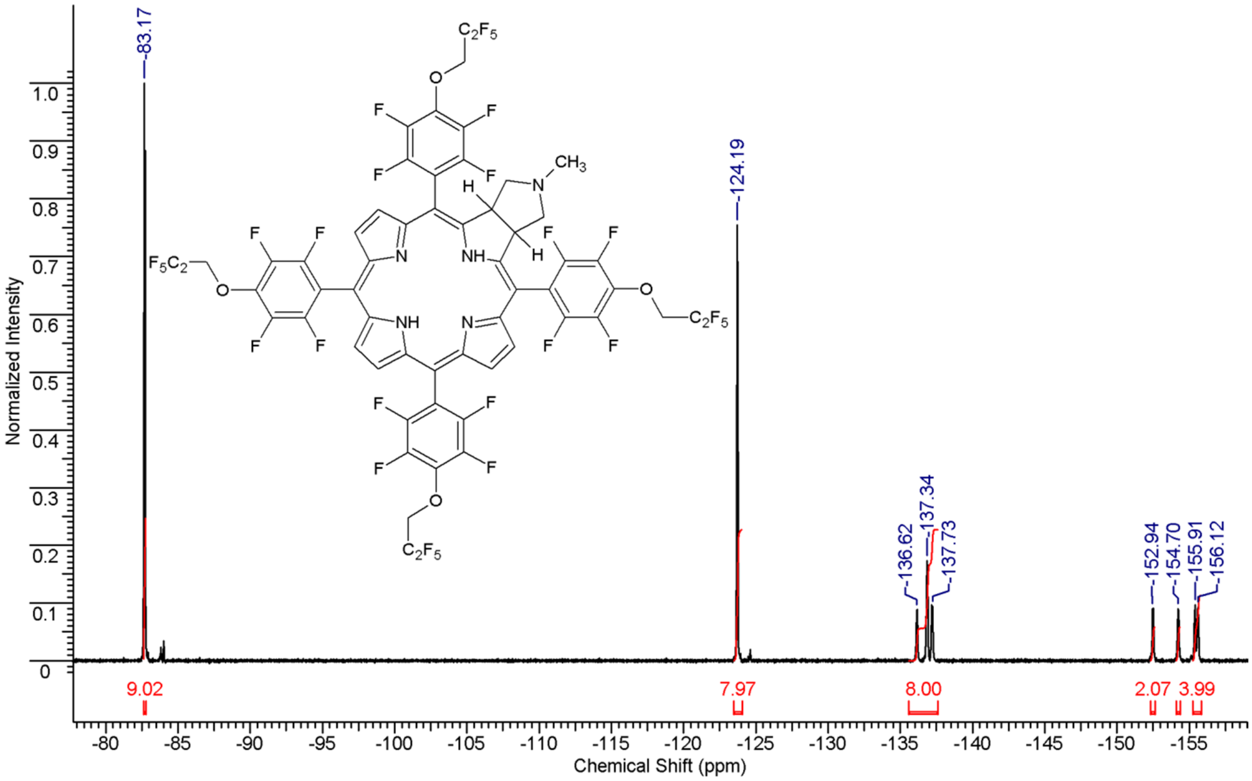

*2,3,3a,21a-tetrahydro-2-methyl-5,10,15,20-tetrakis[2,3,5,6-tetrafluoro-4-(1H,1H-perfluoropentyl -1-oxy)phenyl]-1H,23H,25H-pyrrolo[3,4-β]porphin (**3b**)*

NMR 1H

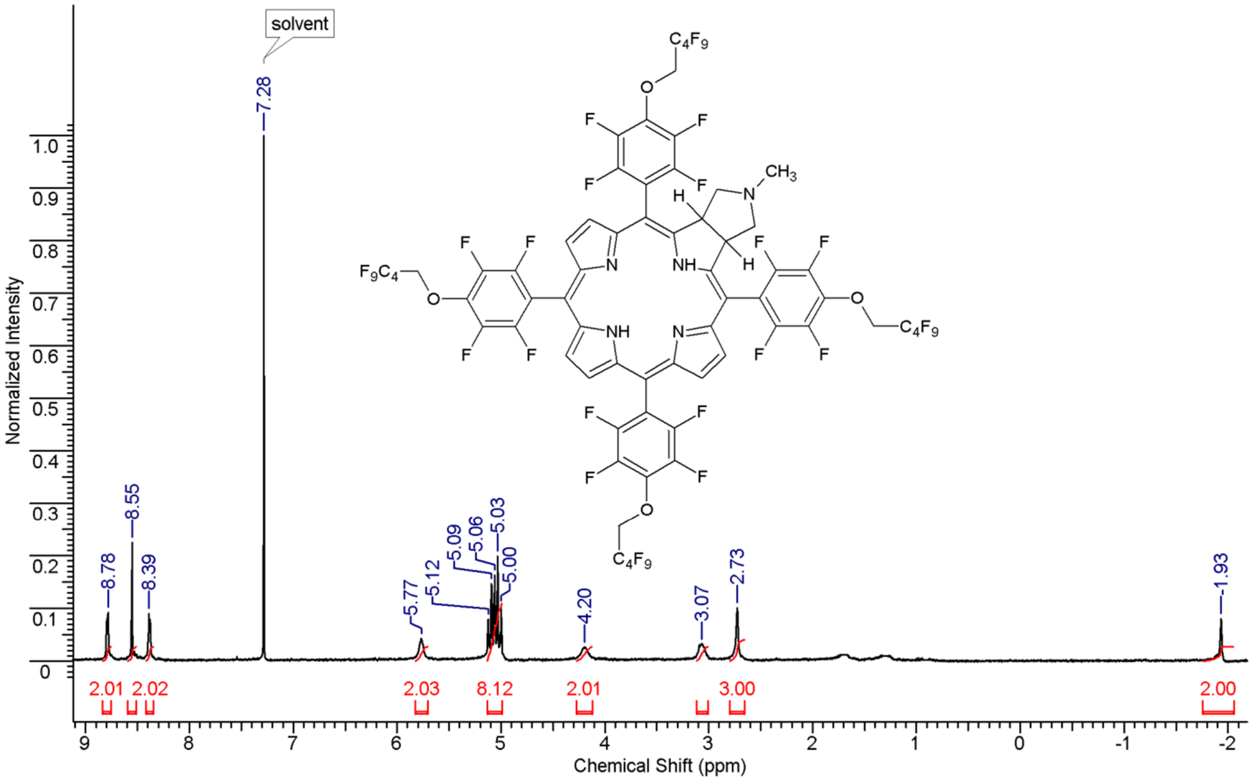

NMR 19F (external standard CF3COOH)

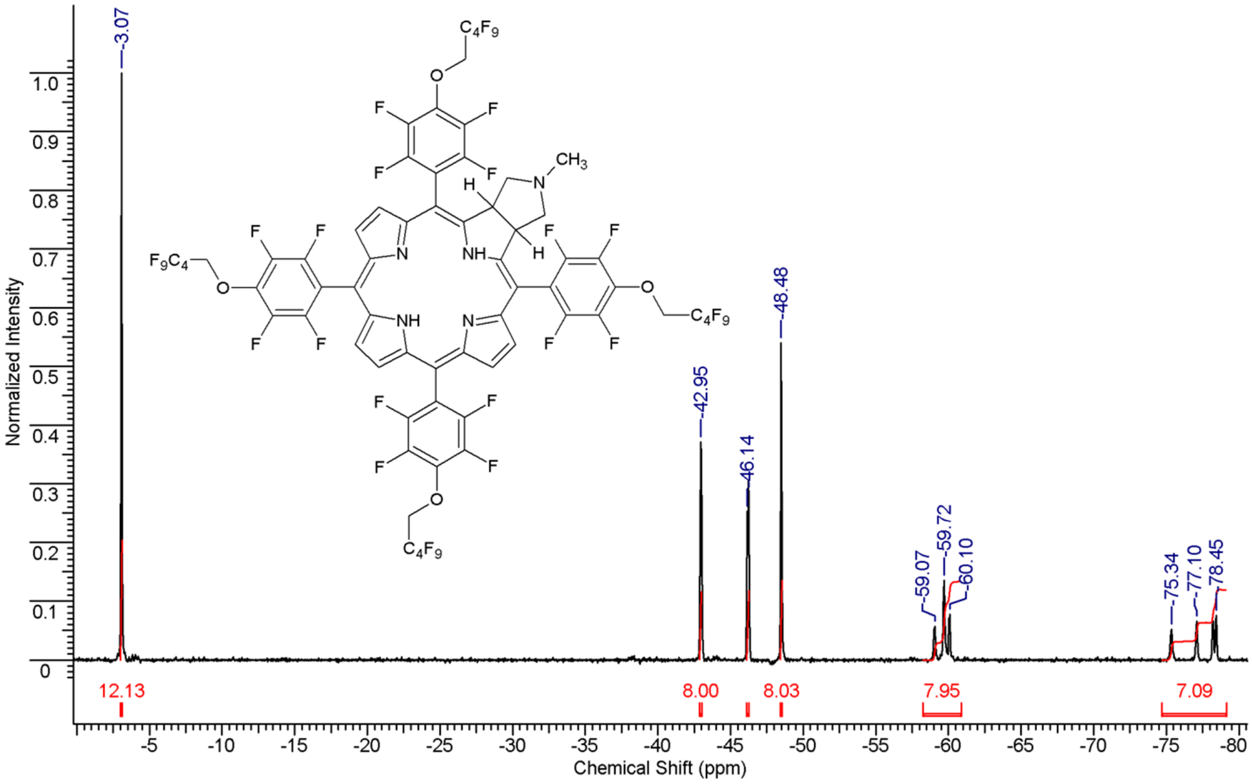

*2,3,3a,21a-tetrahydro-2-methyl-5,10,15,20-tetrakis[2,3,5,6-tetrafluoro-4-(1H,1H-perfluoroheptyl -1-oxy)phenyl]-1H,23H,25H-pyrrolo[3,4-β]porphin (**3c**)*

NMR 1H

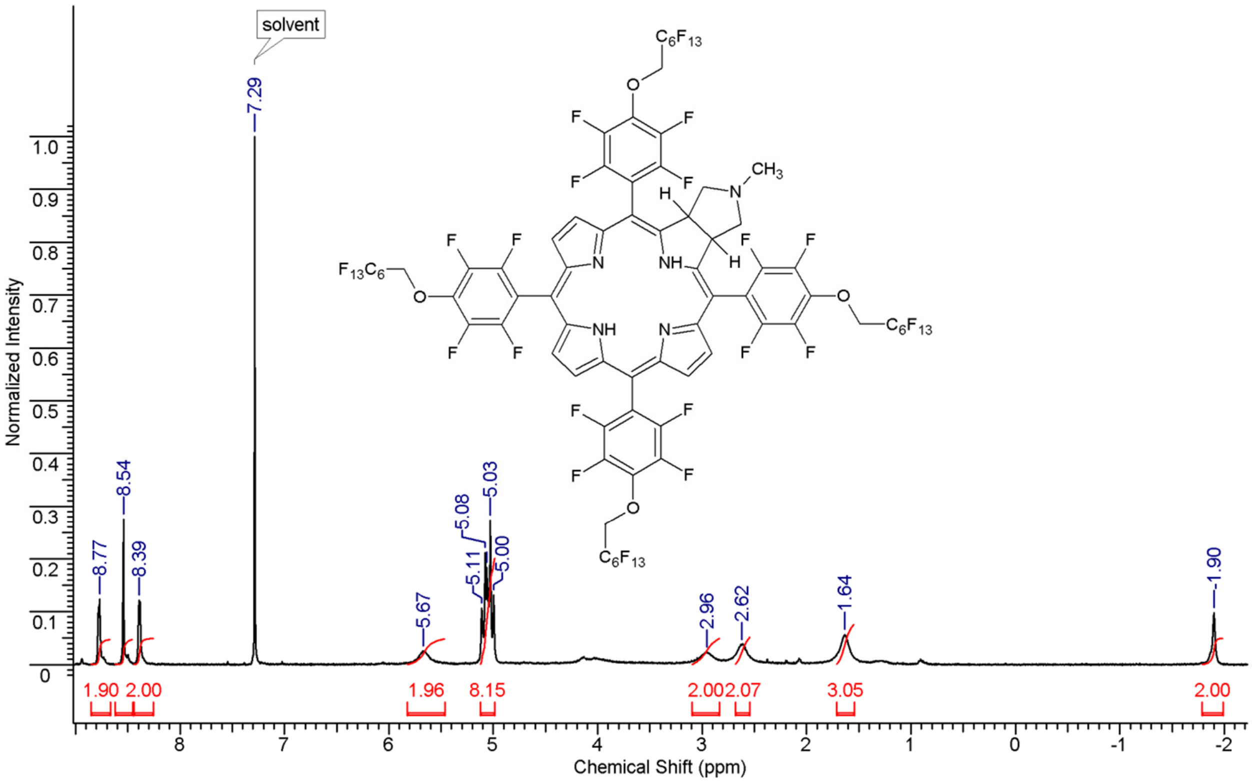

NMR 19F (external standard $CFCl_3$)

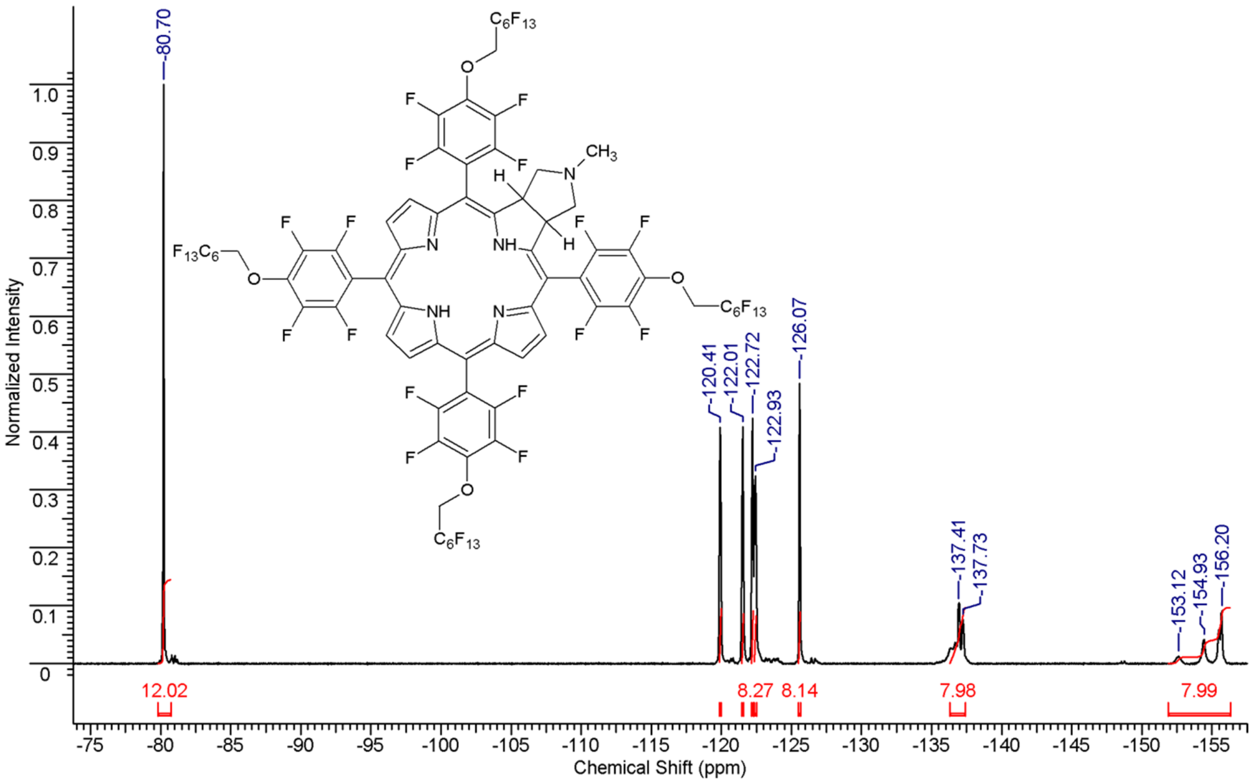

Supplement: Supplementary file 1 [file ijms-24-07995-s001.zip › ijms-2360303-supplementary.pdf]
